# A Narrative Review of u-HA/PLLA, a Bioactive Resorbable Reconstruction Material: Applications in Oral and Maxillofacial Surgery

**DOI:** 10.3390/ma15010150

**Published:** 2021-12-26

**Authors:** Huy Xuan Ngo, Yunpeng Bai, Jingjing Sha, Shinji Ishizuka, Erina Toda, Rie Osako, Akira Kato, Reon Morioka, Mrunalini Ramanathan, Hiroto Tatsumi, Tatsuo Okui, Takahiro Kanno

**Affiliations:** Department of Oral and Maxillofacial Surgery, Shimane University Faculty of Medicine, Izumo 693-8501, Shimane, Japan; ngoxuanhuy158@gmail.com (H.X.N.); xyywq@126.com (Y.B.); jsswjbnjw@gmail.com (J.S.); ishizuka@med.shimane-u.ac.jp (S.I.); et1211@med.shimane-u.ac.jp (E.T.); r.osako@med.shimane-u.ac.jp (R.O.); a-kato0319@med.shimane-u.ac.jp (A.K.); moriokareon@med.shimane-u.ac.jp (R.M.); mru.ramjam2@gmail.com (M.R.); tatsumi@med.shimane-u.ac.jp (H.T.); tokui@med.shimane-u.ac.jp (T.O.)

**Keywords:** oral and maxillofacial surgery, maxillofacial reconstruction, bioresorbability, bioactive osteoconductivity, biocompatibility, uncalcined and unsintered hydroxyapatite, poly L-lactic acid

## Abstract

The advent of bioresorbable materials to overcome limitations and replace traditional bone-reconstruction titanium-plate systems for bone fixation, thus achieving greater efficiency and safety in medical and dental applications, has ushered in a new era in biomaterial development. Because of its bioactive osteoconductive ability and biocompatibility, the forged composite of uncalcined/unsintered hydroxyapatite and poly L-lactic acid (u-HA/PLLA) has attracted considerable interest from researchers in bone tissue engineering, as well as from clinicians, particularly for applications in maxillofacial reconstructive surgery. Thus, various in vitro studies, in vivo studies, and clinical trials have been conducted to investigate the feasibility and weaknesses of this biomaterial in oral and maxillofacial surgery. Various technical improvements have been proposed to optimize its advantages and limit its disadvantages. This narrative review presents an up-to-date, comprehensive review of u-HA/PLLA, a bioactive osteoconductive and bioresorbable bone-reconstruction and -fixation material, in the context of oral and maxillofacial surgery, notably maxillofacial trauma, orthognathic surgery, and maxillofacial reconstruction. It simultaneously introduces new trends in the development of bioresorbable materials that could used in this field. Various studies have shown the superiority of u-HA/PLLA, a third-generation bioresorbable biomaterial with high mechanical strength, biocompatibility, and bioactive osteoconductivity, compared to other bioresorbable materials. Future developments may focus on controlling its bioactivity and biodegradation rate and enhancing its mechanical strength.

## 1. Introduction

Bone reconstruction and bone fixation device systems play crucial roles in the success of oral and maxillofacial surgical treatments. Fundamentally, surgeries involving this region can be divided into two categories: maxillofacial trauma and orthognathic surgery. Traffic accidents and violence often lead to maxillofacial bone fractures [1]. Subsequent treatment requires reconstruction devices to provide steady bone fixation for discontinuous bone fragments, create good osteosynthesis conditions, and restore features to their original size, shape, and location [2]. Various orthognathic-surgery techniques have been instituted to reduce facial-bone-structure abnormalities, improve function, treat chronic disorders caused by these abnormalities, and simultaneously satisfy the patient’s aesthetic demands [3]. After various osteotomy procedures and displacement, with repositioning to the desired position, bone segments must be fixed rigidly to form new bone in the gaps and avoid relapse. Thus, to enhance the quality of oral and maxillofacial surgery, there is a need to develop materials with good mechanical properties, safety, and favorable biological and chemical features for the manufacture of bone-reconstruction and bone-fixation devices.

Devices for bone reconstruction and fixation made of titanium alloys have been extensively used in oral and maxillofacial surgery; they are considered the “gold standard” for rigid fixation. Numerous studies have demonstrated that titanium alloy systems possess properties which are suitable for applications in oral and maxillofacial surgery, such as strength [4], corrosion-resistant oxide layer formation, biocompatibility [5], and osseointegration potential [6]. However, there have also been reports of complications associated with the use of titanium devices. These include infection, pain, hardware exposure, soft tissue erosion, plate palpability over sensitive facial areas, nerve and tooth damage, and cold intolerance [7,8]. Moreover, some studies have demonstrated adverse effects on craniofacial skeletal growth in animals [9,10,11], thus, the use of permanent metallic fixation in pediatric patients requires careful consideration [12]. The presence of metallic hardware may also compromise secondary reconstructive or corrective surgeries, such as bone grafting and osteotomies [13]. Therefore, a second operation is often necessary to remove the titanium devices after bony healing [14,15,16]. For these reasons, it has been necessary to explore alternative materials that can overcome the disadvantages of titanium materials.

With the successful application of bioresorbable suture materials to surgical wound closure [12], the concept of using bioresorbable polymers to manufacture bone-reconstruction and bone-fixation devices emerged in the mid-1960s [17]. The use of bioresorbable materials in facial fractures was first reported in 1971 [18]. Since then, various biological materials have been developed and tested with the aim of identifying an ideal bioresorbable material (i.e., superior to metallic systems). Potential advantages include the lack of a need for further surgery, unrestricted bone growth because of gradual reduction in mechanical strength, lower risk of stress-shielding-related osteoporosis, lack of metallic corrosion-related tissue reaction, and absence of artifacts on computed tomography (CT) [19]. Poly α-esters are the earliest developed, most extensively studied, and most widely used bioresorbable polymers; they have been applied in many disciplines, including oral and maxillofacial surgery [20,21,22,23]. Poly α-esters are high molecular weight aliphatic polyesters with repeating units of α-hydroxy acid (HO-CHR-COOH) derivatives, which are manufactured by ring-opening polymerization [24,25,26]. The resorption of these polymers begins with depolymerization via ester bond hydrolysis. Subsequent metabolism through the citric acid cycle probably involves macrophages and ultimate excretion as water and carbon dioxide [24,27]. Although the strength of bioresorbable fixation devices is poor, it can be increased by using self-reinforcement technology.

Based on the time sequence, composition, physical, chemical, and biological characteristics, the current bioresorbable aliphatic polyester materials can be classified into four principal generations (Table 1):

- First-generation: homogeneous polymers.
- Second-generation: copolymers.
- Third-generation: composites of inorganic/bioceramic fibers or particles, and organic polymers.
- Fourth-generation: composites of inorganic/bioceramic fibers or particles and organic copolymers.

Although polyglycolic acid (PGA), a highly crystalline polymer with superior mechanical properties, was one of the first homogeneous polymers investigated for biomedical applications, there have been few uses for pure PGA in maxillofacial surgery because of its rapid degradation rate [28]. PGA has high tensile strength [29]; however, its degradation time is short, which does not allow complete bone healing. Moreover, the use of PGA has been associated with local inflammation, osteolysis, and sterile abscesses related to the formation of acidic degradation by-products [30,31]. Therefore, pure PGA has limited clinical use as a homogeneous polymer, and is often combined with other resorbable biomaterials.

Polylactic acid (PLA) is a bioresorbable polymer with diverse research and commercial applications because of its inherent biocompatibility, high mechanical strength and modulus, ease of processing, and availability from naturally renewable sources (e.g., corn) [32]. Lactic acid is a chiral molecule that exists as two enantiomers; therefore, PLA has stereoisomers, including poly L-lactide (PLLA), poly D-lactide (PDLA), and poly L/D-lactide (PDLLA) [33]. Among these, PLLA has been used as a first-generation material since the 1990s for fabrication of maxillofacial osteosynthesis devices [34]. PLLA exhibits high tensile strength and low elongation; accordingly, it has a high modulus, making it suitable for load-bearing applications (e.g., orthognathic fixation) [35,36]. However, PLLA has a prolonged degradation period: high molecular weight PLLA may require 2–5.5 years for complete resorption [37,38]. PDLA is another stereoisomeric form of PLA with a slower degradation rate than PLLA; it is also highly biocompatible and can be used in osteosynthetic facial surgeries [39,40]. The copolymer of PLLA and PDLA, known as PDLLA, has lower tensile strength [37]; however, it is stable in orthognathic surgery applications [41,42]. Although PDLLA degrades more rapidly than either PLLA or PDLA [37], the constant degradation rates of these materials have the disadvantages of foreign-body reactions and late-degradation tissue responses caused by their by-products long after the original surgery [37,38]. Such limitations have led to restricted use of these first-generation materials in oral and maxillofacial surgery [23].

Combinations of homogeneous polymers produce copolymers that have the advantages of each constituent polymer, along with significantly improved degradation times. Second-generation bioresorbable materials include copolymers of PGA, PLLA, and PDLA. The most promising copolymer is PLLA/PGA because its strength and resorption rate can be adjusted based on the ratio of PLLA and PGA [43]. For example, the degradation times of 50PLLA/50PGA, 75PLLA/25PGA, and 85PLLA/15PGA are 1–2 months, 4–5 months, and 5–6 months, respectively [37]. These diverse properties of PLLA/PGA facilitate its use in various applications. For example, a copolymer of 82% PLLA and 18% PGA with sufficient strength for 6–8 weeks and a resorption time of 12–18 months [44] is suitable for the manufacture of clinical fixation devices that can be used in midface osteosynthesis [27]. The metabolites of this copolymer (i.e., carbon dioxide and water) are eventually excreted through the lungs [44]. Many studies have indicated that a PLLA/PGA plating system is suitable for oral and maxillofacial surgeries with minimal postoperative complications [27,45,46,47,48].

Despite significant improvements over first-generation materials, copolymers of second-generation materials do not possess any bioactive characteristics [19,49,50,51,52], such as osteoconduction and bone-binding ability, which are crucial to promote faster postoperative bone healing. For example, osteoconduction is the ability of bone-forming cells in the graft implant surface to move across a scaffold and gradually replace it with new bone over time [53]. A mixture of inorganic (bioceramic) fibers or particles and organic polymers to create bioresorbable composites (i.e., third-generation bioresorbable materials) possesses superior features that the previous biodegradable materials lacked (e.g., bioactivity and radiopacity). Two representative composites, u-HA/PLLA (30–40% wt. unsintered hydroxyapatite [u-HA]) and u-HA/PDLLA (70% wt. u-HA), have been used successfully because of their excellent biocompatibility and bone regenerative potential. u-HA/PDLLA is fast resorbing and more brittle; it is suitable for the reconstruction of bone defects caused by trauma or tumors [54,55,56,57,58]. Furthermore, the mechanical strength of u-HA/PLLA facilitates its use in the fabrication of bone fixation systems. u-HA/PLLA has been investigated since the early 1990s and has received considerable attention from researchers and practitioners. Numerous comprehensive and quantitative studies have been conducted to analyze its physical, chemical, and biological properties, evaluate treatment outcomes in oral and maxillofacial surgery, and identify its limitations. Several reports of u-HA/PLLA use in oral and maxillofacial surgery have recently been published. We used these analyses and assessments to provide readers with an up-to-date and comprehensive narrative review of u-HA/PLLA. We also introduce new trends concerning the development of bioresorbable materials in this specialized field.

**Table 1:** Some bioresorbable aliphatic polyester materials used in oral and maxillofacial surgery.

Generation	Name		Structure	Mechanical Strength	Biocompatibility	Bioactive/Osteoconductivity	Degradation Period	Clinical Applications
1	PGA		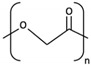 [39]	High [29]	High [59]	− [59]	4–12 months [60]	Tissue engineering. Drug-delivery systems [61]
	PLA	PLLA	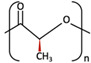 [62]	High [63]	High [64]	− [51]	> 3.5 years [65]	Tissue engineering. Drug-delivery systems. Fixation devices [66]
		PDLA	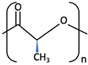 [62]	High [63]	High [64]	− [64]	Longer than PLLA [34]	Tissue engineering. Drug-delivery systems, [66]
		PDLLA	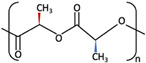 [62]	Lower than PLLA [37]	High [64]	− [64]	12–30 months [37]	Tissue engineering. Drug-delivery systems [66]
2	PLLA/PGA		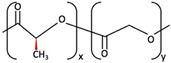 [67]	High [37]	High [68]	− [50]	12–18 months [44]	Tissue engineering. Drug-delivery systems. Fixation devices [66]
3	u-HA/PDLLA		Ca_10_(PO_4_)_6_(OH)_2_ + 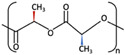	Low [54]	High [55]	+ [56]	12–18 months [69]	Tissue engineering. Fixation devices [70]
	u-HA/PLLA		Ca_10_(PO_4_)_6_(OH)_2_ + 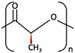	Higher than PLLA [19]	High [19]	+ [19]	> 5 years [49]	Tissue engineering. Fixation devices [70]
4	u-HA/PLLA/PGA		Ca_10_(PO_4_)_6_(OH)_2_ + 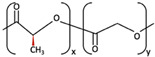	Similar to u-HA/PLLA [71]	High [72]	+ [71]	2–3 years [73]	Tissue engineering. Fixation devices [73]

## 2. u-HA/PLLA, an Outstanding Bioresorbable Composite

Over the past 20 years, u-HA/PLLA has been validated as a bioresorbable material suitable for bone-regeneration applications, particularly in the maxillofacial region. Bioresorbable osteoconductive fixation devices using this material are commercially available, commonly known as super FIXSORB MX or OSTEOTRANS MX, Takiron Co. (Osaka, Japan) initially produced this commercial product; in 2017, its manufacturing was transferred to Teijin Medical Technologies Co., Ltd. (Osaka, Japan). u-HA/PLLA is a composite of u-HA/PLLA particles, the inorganic/bioceramic component, and PLLA, the homogeneous polymer component [19]. This combination allows u-HA/PLLA to utilize the advantages of each element, while alleviating their natural disadvantages [49]. Numerous studies conducted in vitro and in vivo have shown that this bioresorbable material possesses physical, chemical, and biological features superior to other previous materials.

### 2.1. Uncalcined and Unsintered Hydroxyapatite

In the living body, bone is a hierarchically ordered structure with a unique arrangement of two main phases: an inorganic phase that consists of calcium phosphate (CaP) and water (65–70% and 5–8%, respectively) and an organic phase that mainly consists of collagen, a sizeable fibrous protein with a triple-helix structure [74,75,76]. Hydroxyapatite (HA) is the most thermodynamically stable crystalline-phase salt of CaP in the body; it exhibits strong similarity to the mineral component of bone [74,75]. Its chemical formula is Ca_5_(PO_4_)_3_OH, but this is often written as Ca_10_(PO_4_)_6_(OH)_2_ to indicate that the crystal unit cell comprises two molecules. The naturally occurring CaP is calcium-deficient, and is often found as carbonated HA with a Ca/P ratio of < 1.67 [77,78]. It has excellent biocompatibility and bioactivity because of its chemical similarities to the minerals of human bones and teeth [79]; it also exhibits good mechanical strength, porous structure, and osteoconductive, osteoinductive, and osteointegrative characteristics [80]. Therefore, HA has generated considerable interest in various fields of biomedicine. Currently, HA is the biomaterial of choice for multiple medical applications, such as implants or prostheses in orthopedics, maxillofacial surgery, and dentistry, as well as bone restoration or regeneration.

Over the past two decades, multiple HA-synthesis methods have been developed to control its microstructure and particle size. HA preparation methods can be classified into five groups: dry, wet, high-temperature, biogenic, and combination procedures. Details of these methods have been described in various publications [81,82]. The microscopic shape, size, and size distribution of HA particles have been proven to significantly affect their mechanical properties, processing conditions, surface chemistry, biocompatibility, and bioactivity [83,84,85,86,87]. Hence, the method for synthesis of particular HA particles should be selected based on the application.

u-HA is synthesized using the wet method by the hydrolysis of pure calcium hydrogen phosphate anhydride and calcium carbonate, heated in an aqueous solution at 90 °C. After filtration, the particles are incubated at room temperature for 5 h and completely dried for 10 h. The following chemical equation represents the u-HA synthesis:

6CaHPO_4_ + 4CaCO_3_ → Ca_10_(PO_4_)_6_(OH)_2_ + 4CO_2_ + 2H_2_O

The 5–50 μm clusters of the u-HA, aggregated with micro hexagonal prism crystals that had an aspect ratio of approximately 4–10 (length: 2–3 μm; width: 0.3–0.5 μm), were crushed and sieved to create particle sizes in the range of 0.3 to 20 μm (mean: 3 μm). The chemical analysis showed a Ca/P ratio of 1.69, close to the molar ratio of pure HA in a living tissue (1.67). This u-HA contained carbonate (CO_3_^2−^) and particles with medium crystallinity on Fourier-transform infrared absorption spectra and X-ray diffraction. HA in the human bone is not calcined or sintered; its compounds contain some carbonate (CO_3_^2−^) [88]. Therefore, u-HA was similar to the HA in human bone.

Many studies have described the biological activity mechanisms of bioresorbable ceramics in detail [56,89,90,91,92,93,94,95]. In brief, after bioresorbable ceramics are implanted, a layer of nano-apatite crystals formed on the surface of the CaP ceramics absorbs various osteogenic proteins, particularly small molecular proteins (e.g., growth factors) [96,97,98,99]. In a biological environment, the apatite surface layer offers a microenvironment that attracts mesenchymal stem cells to the surface layer; it also induces their differentiation into osteoblasts, while activating and regulating the expression of osteogenic genes in these cells [100,101,102,103]. Various studies [104,105,106,107,108,109] have demonstrated that HAs are disintegrated by osteoclast-like multinucleated giant cells or macrophages [110], but their complete resorption requires substantial time [111]. As mentioned above, HAs synthesized by different methods exhibit distinct properties. HAs synthesized at high temperatures, such as s-HA (sintered at 1000–1400 °C) and c-HA (calcined at 800–900 °C), were reportedly resorbable; however, they are not naturally associated with bioresorbable ceramics and are unavailable for internal bone fixation device applications [112]. In contrast, u-HA has many similarities to human HA, and their natural forms could be similar to each other in vivo [19]. Oonishi et al. reported that u-HA particles were more bioactive and more resorbable, compared with other resorbable bioceramics [113]. Therefore, u-HA was proposed for the fabrication of biocomposites to manufacture bone reconstruction devices.

### 2.2. PLA (PLLA, PDLA, PDLLA)

PLLA, a first-generation biodegradable material, has been utilized in many areas of tissue engineering (e.g., orthopedic fixation devices). PLLA is formed by ring-opening polymerization of monomer L-lactide using stannous octoate and lauryl alcohol as initiator and polymerization modulator, respectively; this is followed by extraction using ethyl acetate, as described in previous studies [114].

Compared to other first-generation biomaterials (i.e., PGA, PDLA, and PDLLA), PLLA possesses superior features, such as greater tensile strength and modulus. However, the resorbable plates made from these polymers do not provide sufficient stability to counteract the masticatory forces, leading to higher rates of delayed union, compared to titanium-based devices [115,116]. Consequently, increased thicknesses of PLLA plate systems are required to approach the strengths of titanium plate systems, which leads to problems such as increased palpability [117]. However, its slower degradation has been presumed to reduce inflammatory tissue reactions [118,119]; thus, it has robust biocompatibility and potential bioresorbability. Some investigations [37,38] have demonstrated that PLLA devices require 2–5.5 years to resorb completely. Slow resorption is a critical disadvantage and leads to several complications, including late aseptic swelling [120,121], osteolytic changes at the implant site [119,122], and late-degradation tissue response [65]. Bergsma et al. [65] suggested that the aseptic swelling and the tissue response might be caused by a change in PLLA morphology during material degradation. In the early stages after implantation, PLLA degradation is probably an extracellular hydrolytic process. After PLLA has degraded into fragments and particles with higher crystallinity, it becomes stable and resistant to hydrolysis. These fragments or particles induce a cellular reaction and are internalized by phagocytes in membrane-bound vacuoles. Although phagocytes, particularly macrophages, release many lysosomal hydrolytic enzymes (e.g., acid phosphatase and lactic dehydrogenase) to degrade PLLA, these enzymes cannot actively digest PLLA particles. Therefore, PLLA particles either persist in the intracellular environment or are egested into the extracellular space. These indigestible foreign-body particles may cause a continuous inflow of macrophages that may repeatedly phagocytose the PLLA particles and re-initiate the intracellular cycle. This phenomenon induces and maintains clinically significant swelling for substantial intervals after PLLA device implantation.

PLLA was used to pioneer the applications of bioresorbable materials in oral and maxillofacial surgery because of its good mechanical features and biocompatibility. However, the presence of inflammatory reactions long after implantation has been an important problem. Furthermore, incomplete restitution of the medullary canal after implant resorption [34,123] and early micromovement of the implant [124,125,126] were also reported. This showed that PLLA could not accelerate bone healing or osteoconductive capacity, which are vital processes for the reconstruction of critical defects in maxillofacial bones. Therefore, PLLA is no longer used in oral and maxillofacial surgeries.

### 2.3. Forged Composite of u-HA Particles and PLLA Polymer

Tiny granules of uniformly distributed u-HA microparticles within a PLLA matrix were obtained by precipitation of the polymer solution that had been formed by dropwise addition of ethanol to a PLLA/dichloromethane solution. The granules were extruded to create a thick billet, which was then forged at 103 °C into a thin billet without fibrillation using a compression molding process. Finally, it was cut using a lathe into devices with different shapes and sizes (e.g., screws, pins, and plates). Its deformation ratio (i.e., percentage of cross-sectional area between thick billet and thin billet) was established at a value of 2.8 with respect to the mechanical strength and two-dimensional balance [19]. The resulting composite had the advantages of each component, including the osteoconductivity of u-HA, the strength of PLLA, and the biocompatibility and bioresorbability of both components; it also exhibited other outstanding features, such as improved mechanical properties and radiopacity. Therefore, the emergence of u-HA/PLLA marked a critical event in the development of bioresorbable materials.

Osteoconductivity and bone-bonding are unique characteristics that make u-HA/PLLA material superior to previous types of bioresorbable polymers. In an in vitro study, Shikinami et al. [19] observed that numerous apatite crystals began to form on the composite surface in simulated body fluid at 37 °C within 3–6 days; these crystals covered the entire surface in a thick layer in 7 days. The results suggested that apatite deposition might lead to early bonding between device and bone in vivo. In another study [127], Yasunaga compared the bond strengths and behaviors of these u-HA/PLLA composites on the surface of bone cortex in vivo. Direct contact between the bone and composite plates was clearly observed after implantation, but no bonding was present between the bone and the PLLA plates. These findings indicate that u-HA/PLLA possesses superior bone-bonding features, compared to PLLA. In addition, Dong et al. [51] demonstrated that the amount of newly formed bone in a rat model was significantly greater in the u-HA/PLLA group than in the PLLA group. The osteoconductive features of u-HA/PLLA were also demonstrated in an in vivo study by Ikumi [128]. Critical bone defects (8 mm in diameter) were surgically created on the parietal bones of rats and covered by u-HA/PLLA or collagen membranes. The bone defects were evaluated using micro-CT and histological analysis at 2 and 4 weeks postoperatively. The results at 4 weeks postoperatively showed that bone growth was significantly greater in the u-HA/PLLA group than in the collagen membrane group. Notably, the u-HA/PLLA membranes used in that study had been treated with ultraviolet (UV) radiation to improve biocompatibility. Ikawa also suggested that UV irradiation enhanced the regenerative potential of the u-HA/PLLA mesh [129]. Moroi et al. suggested that UV treatment increases the hydrophilicity of u-HA/PLLA, leading to a greater proportion of u-HA granules in this composite; it also allows easier u-HA exposure on the subsurface, leading to enhanced osteoconductivity [130]. Because of these superior abilities, u-HA/PLLA material offers outstanding benefits for clinical applications (e.g., direct bone-bonding-related reinforced stability after implantation), and thus, promotes bone defect healing. Importantly, first- and second-generation bioresorbable materials do not offer such benefits.

**Figure 1:**
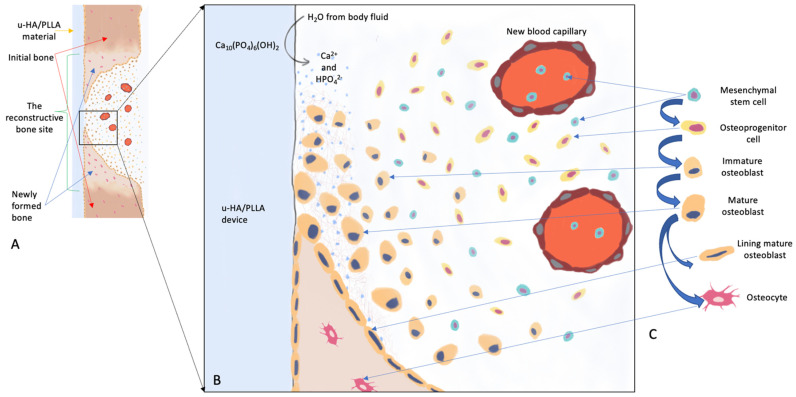
Schematic diagram representing the bioactive/osteoconductive mechanism of the u-HA/PLLA device. (**A**), The u-HA/PLLA device covers the space between two bone fragments. (**B**), Magnified view of image A shows events that occur at the lower margin of the bone fragment. After implantation, body fluids diffuse more easily into the composite devices and cause the dissolution of u-HA particles into surrounding areas. This increases the concentrations of calcium and phosphate ions in the local environment, thus leading to a sequence of events that facilitate new bone formation around the material. This sequence of events includes promoting mesenchymal stem cell migration to the surface layer, inducing mesenchymal stem cell differentiation into osteoblasts, and activating/regulating the expression of osteogenic genes in mesenchymal stem cells. (**C**), Progression of osteoblast differentiation from mesenchymal stem cells.

Theoretically, u-HA/PLLA material is biocompatible and bioresorbable because both of its components possess these features. Indeed, an in vivo study investigating the biodegradation of this material over 1 year [131] showed no inflammatory cells (e.g., macrophages or multinucleated giant cells) around the implants. However, its biodegradation process differs from the processes of previous bioresorbable materials. As mentioned above, the degradation of PLLA and other polymers occurs by hydrolysis, while the degradation of u-HA particles occurs by phagocytosis. Therefore, the combination of u-HA and PLLA requires both hydrolysis and phagocytosis for degradation [19]. Because no chemical bond is present between u-HA and PLLA and the composite exhibits surface porosity, body fluid can diffuse more easily into these composite devices [131]. According to Shikinami et al. [49], during the early stage after implantation, partial dissolution of CaP ceramic macrocrystals increases the concentrations of calcium and phosphate ions in the local environment. The subsequent sequence of events (e.g., mesenchymal stem cell migration to the surface layer, induction of mesenchymal stem cell differentiation into osteoblasts, and activation and regulation of osteogenic gene expression in these cells) leads to new bone formation around the material. Bioactive and bioresorbable u-HA particles are then resorbed into the surrounding natural bone, where they show strong osteoconductivity without the onset of physical irritation (Figure 1). The resorption of u-HA particles on the surface of the porous composites leads to increased water uptake, which may accelerate PLLA hydrolysis [131]. The degradation of the PLLA component then occurs as described above. Therefore, the degradation time of this material continues to be prolonged (i.e., 4–5 years after implantation) [49]. Although most long-term in vivo studies [132,133] have detected no adverse tissue reactions with the material, the risk of a harmful reaction increases during prolonged retention.

As mentioned above, although some PLLA devices exhibit high mechanical strength, this strength is sometimes insufficient to counteract masticatory forces [115,116]. However, the combination of u-HA particles and PLLA polymer results in a novel material with improved mechanical properties. An in vitro study by Shikinami et al. [19] compared 10 mechanical parameters (e.g., bending strength, tensile strength, compression strength, impact strength, shear strength, and modulus) among devices made using only PLLA and devices made using composites of u-HA and PLLA with various weight percentages of u-HA (from 20% to 50%). The results showed that u-HA significantly enhanced the mechanical strength of u-HA/PLLA composites, compared to PLLA polymers. These composites exhibited the greatest potency in all mechanical properties. For example, the bending strength of approximately 270 MPa was far greater than the bending strength of cortical bone, while the modulus of 12 GPa was equivalent to the modulus of cortical bone. Furthermore, the u-HA/PLLA material maintained 85% and 80% of its initial bending strength after 8 and 25 weeks in the subcutaneous tissue, respectively [131]. This mechanical strength persisted for a longer interval, compared with previous bioresorbable materials [134,135,136]. Generally, the union of a fractured bone requires 8–12 weeks of fixation; therefore, the composite was suitable for bone healing and could be used in fixation device manufacturing.

In addition to the characteristics mentioned above, u-HA/PLLA material possesses other features that were absent from previous materials. Importantly, because of its greater strength, the thickness of u-HA/PLLA devices is reduced; this decreases palpability in areas covered by thin skin. Furthermore, u-HA/PLLA miniplates are easily bent to adjust to the bone contour using a water bath (75 °C). It is also easier to apply in areas with complex anatomy. Finally, it is easier to visualize u-HA/PLLA devices on X-rays and CT scans because of the radiopaque u-HA particles.

In conclusion, based on the evidence from in vitro and in vivo studies of u-HA/PLLA, this third-generation biomaterial completely outperforms previous biomaterials and may be useful for manufacturing bone reconstructive devices that can be used in clinical practice.

## 3. Clinical Applications of u-HA/PLLA in Oral and Maxillofacial Surgery

Based on anatomy and function, the maxillofacial bones can be divided into three main parts: the upper face (including orbital regions), the midface (primarily comprising maxillae and zygomatic bones), and the lower face (consisting of the mandible) [137]. Each part has unique skeletal features that ensure its proper function. For example, the mandible is the largest, strongest, and only moveable bone of the facial skeleton. Its functions include chewing, swallowing, and speaking; many strong muscles of mastication (e.g., masseter and medial pterygoid) attach to it to perform functional movements [138]. The fixation of the mandible is more complicated because of multidirectional pulling forces. This requires fixation devices to have sufficient strength for fragment immobilization to facilitate bone healing; they must also allow specific mandibular movements postoperatively to ensure daily nutrition. To achieve good outcomes in this area, many requirements must be considered with respect to the materials that are used to fabricate facial-bone-reconstruction and bone-fixation devices.

**Figure 2:**
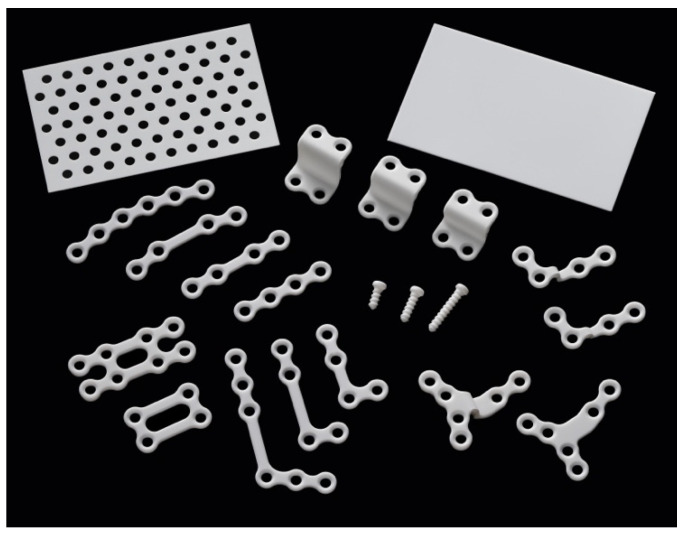
OSTEOTRANS MX system manufactured by Teijin Medical Technologies Company. The plates have diverse shapes with thicknesses of 1.0–1.4 mm. The screws are available in two diameters, 2.0 mm and 2.7 mm, with lengths of 4–16 mm and 16–20 mm, respectively. The square meshes/sheets measure 25 × 50 mm^2^ or 50 × 50 mm^2^ with a thickness of 0.3–0.7 mm.

Bioresorbable-device systems made from u-HA/PLLA composite are currently used in a wide range of surgical fields, such as orthopedic surgery, oral and maxillofacial surgery, plastic and reconstructive surgery, neurosurgery, and thoracic surgery. Products applied in oral and maxillofacial surgery include plates, screws, and meshes or sheets with different shapes, lengths, and sizes suitable for the diverse requirements of maxillofacial bone structure (Figure 2). The percentages of u-HA particles in the screws and plates are 30% and 40%, respectively. This section presents the clinically proven effects of this bioresorbable material in two main categories: maxillofacial trauma and orthognathic surgery (Table 2).

### 3.1. Maxillofacial Trauma

#### 3.1.1. Orbital Wall Fractures

The ideal implant material for orbital reconstruction must be biocompatible, noncarcinogenic, nonallergenic, radiopaque, sufficiently strong (to support orbital contents in patients with large orbital wall defects and large bony fragment dislocations), and easy to manipulate. It should also be cost-effective and amenable to sterilization [148]. Before u-HA/PLLA composites, diverse autogenous and alloplastic implant materials were used for the reconstruction of orbital walls. Autogenous bones (e.g., ilium, calvarium, rib, maxilla, and mandible) are well tolerated but are difficult to adapt to the shape of the defect; they also require a donor site surgery [149]. Alloplastic materials can be divided into nonresorbable materials (e.g., titanium mesh [150,151], porous polyethylene [Medpor] [152,153], silicone elastomers [154], porous polyethylene sheet [155], and HA [156]) and resorbable materials (e.g., polycaprolactone [157], polydioxanone [158], gelatin film [Gelfilm] [159], and PDLLA [160]). However, each material has unique limitations, including manipulation difficulties, poor visualization on X-rays and CT scans, implant extrusion, high infection rates, or high costs [139,157,161,162]. Based on the characteristics demonstrated during in vitro and in vivo studies, u-HA/PLLA is a promising biodegradable material for orbital reconstruction because it fulfills these clinical requirements. Indeed, many clinical investigations have been conducted to evaluate the efficacy of u-HA/PLLA in this regard.

An important benefit of u-HA/PLLA sheets is their visibility on radiographs and CT scans, because they appear brighter than the previously used resorbable implants [160]. Because the radiation exposure during CT examination is insufficient to cause damage, Tsumiyama suggested that CT scans may be used regularly [161]. The easy observation of this material on radiographs is helpful for assessing changes in physical properties, biocompatibility, bioactivity, and biodegradability; it also helps detect complications during long-term follow-up.

In a 2015 study by Park et al., 10 patients with pure medial wall blow-out fractures and defects > 2.5 cm^2^ underwent repair using u-HA/PLLA devices. CT scans and plain radiographs were used to measure changes in implant position and shape to analyze the rigidity of u-HA/PLLA. The results showed no statistically significant differences in implant position and shape, either immediately or at 2 months postoperatively. The study concluded that u-HA/PLLA implants are physically difficult to manage and mold; they are also inconvenient for use in reconstructing a complex three-dimensional (3D) defect. However, u-HA/PLLA devices can be formed by immersion in warm water [139]. In another study by Kohyama, 70 patients with orbital wall fractures—treated surgically using u-HA/PLLA sheets)—were followed up for durations of 3.3–52.3 months using CT scans. Measurements of bony orbital volumes immediately postoperatively and on the latest follow-up CT scans demonstrated that the u-HA/PLLA sheets firmly maintained their fit to the orbital wall contours without deviation, soft-tissue herniation, broad or round sagging effects, or other changes. These results confirmed the desirable handling characteristics, initial mechanical strength, and long-term structural stability of this material; they suggested that u-HA/PLLA is biocompatible and osteoconductive [162]. In another study, Hess area ratio assessment of multiple orbital fracture repairs with different implant materials showed absorption of the u-HA/PLLA sheet and replacement with calcification on long-term follow-up scans, which is advantageous in critical defect treatment [163].

In a study by Kohyama, no patients exhibited any sign of infection or inflammation, although the implants are usually connected to the walls of the nasal cavity or paranasal sinuses [162]. Similar findings in studies by Tsumiyama et al. [161] and Jang et al. [164] confirm the biocompatibility of this material. These studies have described some postoperative complications, including diplopia in the extreme gaze, enophthalmos, infraorbital nerve disturbance, extraocular muscle palsy, and severe trigeminal nerve palsy [163,164,165]. However, these complications may be explained by fracture extension and severity [162]. Some authors have presumed that postoperative enophthalmos is more closely related to surgical skills than to the intrinsic rigidity or durability of the implant [139].

**Figure 3:**
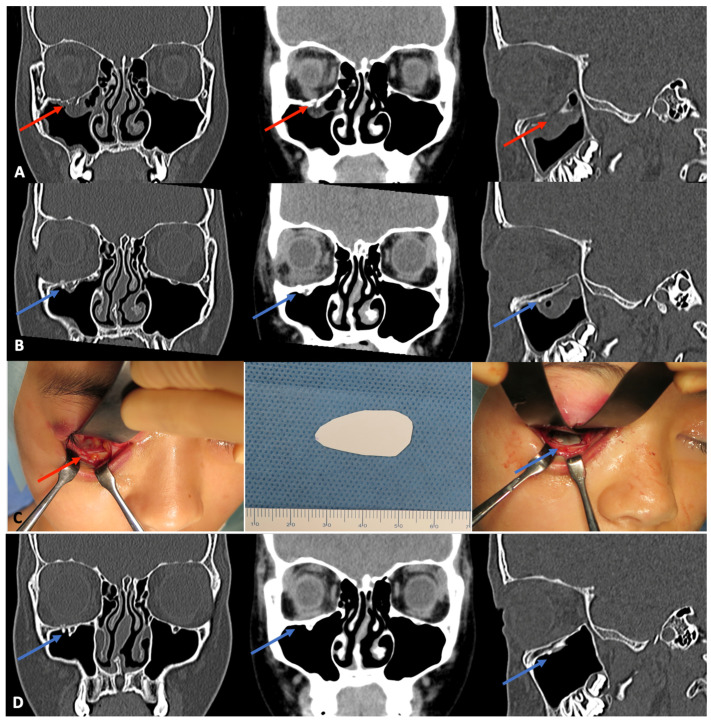
Example of navigation-assisted orbital fracture reconstruction using u-HA/PLLA composite sheets and tack fixation system. A 29-year-old woman was diagnosed with a blow-out fracture of the right orbital floor, which resulted in orbital content herniation. (**A**), Preoperative CT scan images in the coronal and sagittal planes show the right orbital floor fracture and the orbital contents that had prolapsed into the right maxillary sinus (red arrows). (**B**), Immediate postoperative CT scans in the coronal and sagittal planes show the u-HA/PLLA sheet covering the orbital floor fracture (blue arrows). (**C**), Intraoperative view of the orbital floor fracture after surgical exposure. The prepared u-HA/PLLA sheet and tack screw were used to cover the fracture. (**D**), One-year postoperative CT scan shows the completely healed right orbital floor (blue arrows). The u-HA/PLLA sheet remained visible, and there was no evidence of orbital contents in the right maxillary sinus.

In 2016, Kanno and colleagues [166] reported navigation-assisted orbital fracture reconstruction using u-HA/PLLA composite sheets with a tack fixation system (0.5-mm-thick panel sheets and 5-mm-long tacks with a very low screw-head profile) (Figure 3). The u-HA/PLLA sheets were preoperatively fabricated, shaped, and prepared using a computer-assisted 3D morphological customization technique, mirroring the preoperative 3D model image of the u-HA/PLLA sheet. This technique reduced the risk of sheet migration in the orbit, while providing excellent stability for orbital wall reconstruction at fragile and anatomically complicated periorbital maxillofacial bony regions. This avoided the risk of unexpected orbital wall breakage, which could exacerbate the orbital wall defect and cause screw loosening. In addition, an intraoperative optical navigation system based on preoperative CT data was used to determine the extent of orbital wall defects and confirm accurate 3D placement of the u-HA/PLLA implants for reconstruction. A combination of the u-HA/PLLA composite sheet with tack fixation and intraoperative navigation systems provides satisfactory ophthalmic functional results with respect to large and complex combined orbital floor and medial wall fracture reconstruction; it avoids intraoperative or postoperative complications throughout 6 months of follow-up. These results have attracted considerable attention from clinicians and researchers, with numerous studies having been conducted to evaluate the effectiveness of this surgical technique [165,166,167,168,169,170]. An assessment of the accuracy achieved in patients undergoing orbital reconstruction for orbital floor defects using a u-HA/PLLA composite system, with and without intraoperative navigation [167], showed that one patient had persistent slight sursumversion diplopia after surgery in the non-navigation group; mean reconstructed orbital volume accuracy differed significantly between groups. Hence, complex orbital reconstruction using an optimal bioactive material with intraoperative navigation is an accurate and reliable method that avoids the high costs and long preparation times needed for implant fabrication. It is suitable for use in emergencies, as well as perioperative positional evaluation. Although the sample sizes were small in these studies, subsequent investigations have confirmed these findings.

Hwang [171] described a 35-year-old woman with left tripod fracture, left orbital medial wall and floor fracture, and left superficial lateral palpebral ligament rupture; the patient had enophthalmos of the left eye at approximately 2 years after surgery with u-HA/PLLA mesh. The mesh was removed during secondary orbital reconstruction using an iliac bone graft. Gel permeation chromatography and Fourier-transform infrared spectroscopy analyses suggested that u-HA/PLLA had favorable degradation properties. As mentioned above, the degradation time of the new materials ranges from 4 to 5 years after implantation [49]. Most relevant human studies have used short follow-up intervals, during which the u-HA/PLLA devices remained detectable on radiographs. Hayashi [172] described a patient who was followed up for 60 months after fracture surgery that had involved u-HA/PLLA devices. In that patient, the device had begun to assimilate at 1 year postoperatively, and was almost fully absorbed at 5 years. Theoretically, extended material persistence after implantation is associated with a greater risk of complications, regardless of whether the material is biocompatible. However, there have been no published reports concerning direct complications of u-HA/PLLA devices used for this particular treatment.

#### 3.1.2. Midfacial Fractures

**Figure 4:**
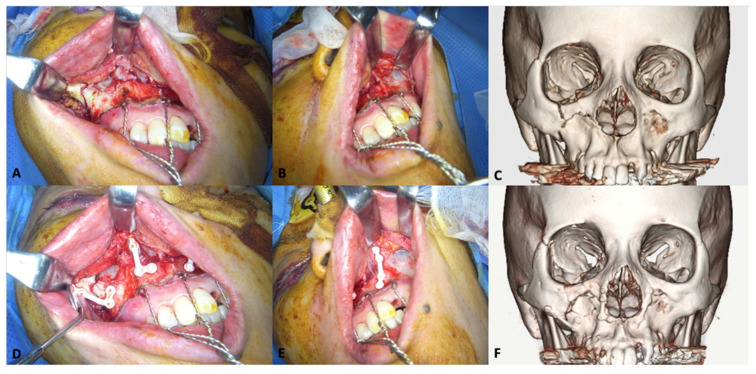
Representative clinical photographs of the u-HA/PLLA fixation system in the midfacial region. A 55-year-old woman exhibited complex maxillofacial fractures, including Le Fort I maxillary fracture, zygomaticomaxillary complex fracture, and orbital floor fracture on the right side, and maxillary sinus fracture on the left side. The Le Fort I maxillary fractures were fixed using u-HA/PLLA plates. (**A**), Complex fractures on the right side of the maxilla. (**B**), Maxillary fracture on the left side. (**C**), Preoperative CT scan image. (**D**), Two L-shaped u-HA/PLLA plates were used to fix fractures on the left side. (**E**), A u-HA/PLLA straight plate was implanted to correct the fracture on the left side. (**F**), Six-month postoperative CT scan image.

Titanium fixation systems have been widely used in the surgical treatment of maxillofacial fractures because of their strength, ease of handling, dimensional stability [173], minimal scatter on CT scan, and obvious visibility on radiography and magnetic resonance imaging [174]. However, they have several disadvantages, such as potential interference with facial growth [11], thermal sensitivity [174], plate migration [175], and interference with diagnostic imaging [176]. Moreover, long-term interactions between this device system and living tissues may cause cortical bone osteopenia through efforts to protect against stress and corrosion [118]. Therefore, the superior properties of u-HA/PLLA are considered suitable for osteofixation surgery in the midfacial area (Figure 4).

In 2013, Hayashi [172] conducted a study to assess the effectiveness of surgical treatment for facial fractures using u-HA/PLLA composite devices in 17 patients with frontal or zygomatic bone fractures. Intraoperatively, the miniplates could slowly be bent by ≤ 60 degrees using forceps at room temperature, or by >60 degrees through immersion in hot water at a temperature of 65–68 °C for 10–60 s. The postoperative follow-up period in that study was 6–60 months. All fractures healed well, and in one patient, the device was almost completely absorbed at 5 years postoperatively. These findings suggested that the degradation time of this material remained excessively prolonged. There were also two reported complications in separate patients: excess frontal bone formation at 6 months postoperatively without any pain or infection (n = 1) and subcutaneous swelling in the upper eyelid at 2 years postoperatively (n = 1). Hayashi suggested that because late PLLA degeneration requires an enzymolysis reaction, the remaining PLLA could cause infection. Despite the reported complications, u-HA/PLLA was considered beneficial because of its strength, thinness, and radiopacity.

Landes [140] evaluated 29 patients with malar and midfacial fractures who underwent surgical treatment using u-HA/PLLA composite fixation devices. The findings demonstrated good intraoperative handling, with bending of ≤40 degrees angulation at room temperature. During the follow-up interval of 12–67 months, all fractures successfully stabilized and re-ossified without any instances of nonunion, although two patients experienced foreign body reactions. In these two patients, local redness and swelling were observed at 15 and 33 months after fracture fixation, although plate removal led to symptom resolution. However, Landes reported that the implants were palpable in all patients after surgery. The noticeable residual prominence that remained on long-term follow-up examinations could have been newly formed bone that had integrated and partially substituted the u-HA/PLLA plate. The findings indicated that u-HA/PLLA material provided reliable and satisfactory internal fixation, intraoperative handling, long-term stability, and biocompatibility.

Sukegawa et al. [142] also reported similar findings in a clinical study. Of 35 patients with maxillary and zygomatic fractures, all 14 treated using u-HA/PLLA devices had good fracture stabilization and re-ossification with few complications. One patient with a zygomatic fracture had discomfort at the infraorbital rim (without pus discharge) at 10 months postoperatively. As the strength of the material approached the strength of titanium plates, the thicker plates were presumed to increase the risk of palpability, particularly in areas with thin skin, thus causing discomfort to the patient. After a few months without any improvement, the plate was removed.

Kim et al. published two papers in 2019 concerning the stability and aesthetic outcomes after one-point fixation of zygomaticomaxillary complex fractures using u-HA/PLLA [141] and comparing degradation patterns between PLA/PGA and u-HA/PLLA [177]. For patients with mild to moderate displacement of zygomaticomaxillary fractures, one-point fixation at the zygomaticomaxillary buttress region using u-HA/PLLA plates was less invasive, maintained sufficient stability, and demonstrated promising results. This technique also avoided complications (e.g., wound infection, plate exposure, bony nonunion, and incision scars) because of the high mechanical strength, biocompatibility, and osteoconductive ability of this material. All patients in the study were satisfied with the symmetric soft tissue malar appearance at 3 months postoperatively, and with bony stability and symmetric bony malar appearance at 6 months postoperatively [141]. Through direct comparison in one patient [177], Kim et al. reported the differences in the degradation patterns between PLA/PGA and u-HA/PLLA materials. For PLA/PGA, there was persistent swelling and redness on the left side of the face at 2 years after implantation; the plate was later surgically removed. Solid, tiny remnants were also found near the surgical site. The same patient subsequently had a zygomaticomaxillary fracture on the right side; thus, the patient underwent internal fixation surgery using u-HA/PLLA composite material. Contrary to PLA/PGA, swelling and redness only appeared after a traumatic injury to the right cheek; u-HA/PLLA plate remnants were observed as small powder-like particles during the removal surgery. Based on these findings, Kim et al. concluded that u-HA/PLLA plates might be ideal absorbable materials for use in facial surgeries.

#### 3.1.3. Mandibular Fractures

**Figure 5:**
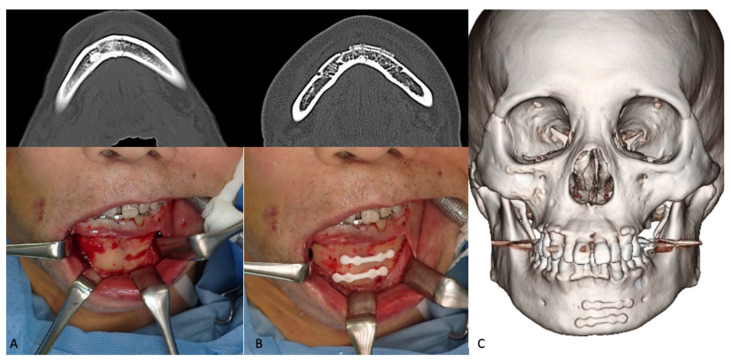
Representative photographs of the two-miniplate fixation technique for mandibular symphysis fractures using the u-HA/PLLA bone-fixation-device system. An 83-year-old man was diagnosed with right orbital floor, right zygomaticomaxillary, and mandibular symphysis fractures. The mandibular fracture was treated using two straight u-HA/PLLA plates. (**A**), **Upper**, Preoperative CT scan image; **Lower**, Exposed mandibular fracture. (**B**), **Upper**, Postoperative CT scan image; **Lower**, Mandibular fracture fixation using two u-HA/PLLA miniplates. (**C**), Six-month postoperative 3D CT scan.

In 2016, Sukegawa et al. [142] reported the clinical evaluation of u-HA/PLLA composite devices used for the internal fixation of mandibular fractures in 21 patients. A 2-miniplate technique was used for parasymphysis, symphysis, and body fractures, i.e., 1.0-mm or 1.4-mm plates and ≥2 screws were used on each side of the fracture (Figure 5). Postoperatively, guiding elastics were used to manage occlusion. The fracture lines healed completely in all patients without any foreign body reactions, and the mechanical strength of the u-HA/PLLA composite devices was sufficient for rigid fracture fixation. However, thicker u-HA/PLLA plates were used to increase the strengths of the devices, which increased the risk of exposure. Plate exposures were observed in the para-symphysis and body of the mandible in two patients, suggesting that it is crucial to sufficiently cover the fixation devices by means of the oral vestibular approach. Lee et al. [178] performed an assessment of outcomes and complications of mandibular fractures treated using u-HA/PLLA fixation systems in 11 patients; their findings demonstrated that this system has sufficient strength for the treatment of mandibular fractures. Moreover, they noted that the greatest advantage of this material was that it did not require a second plate-removal surgery. Moreover, in a 3D analysis of 40 patients with mandibular body fractures that had been treated with open reduction and internal fixation using either a titanium or u-HA/PLLA fixation device, the stabilities of u-HA/PLLA plates/screws and titanium miniplates/screws were equivalent for ≤6 months postoperatively [179]. The results supported the use of u-HA/PLLA as an alternative to titanium alloys for the internal fixation of mandibular fractures.

Endoscope-assisted open reduction and internal fixation of mandibular sub-condylar fractures has been reported as a minimally invasive procedure with few complications [180]. In 2017, Son et al. [181] evaluated the stability and efficiency of the u-HA/PLLA composite system in 11 patients with mandibular sub-condyle fractures that had been treated endoscopically. They observed complete bone formation around the fracture (fading of the fracture lines) with no change in fractured segment position. They also observed increased radiopacity around plates and screws on 3-month postoperative cone-beam CT scans in all patients. Calcification around the devices was presumably caused by the osteoconductivity of the u-HA component of the hardware. Postoperatively, elastic bands were used to limit mandibular movement and reduce occlusal interferences from condylar swelling for 1 week, except in two patients who required postoperative intermaxillary fixation. The patients began mouth opening exercises at 3 weeks postoperatively and achieved the full opening range by 6 weeks. This indicated that u-HA/PLLA bioresorbable plates provided sufficient strength during fracture healing. The study reported mild swelling and temporomandibular joint pain in two patients, which subsided after treatment. A study by Kim [182] analyzed 28 patients with mandibular sub-condylar fractures that had been treated endoscopically using u-HA/PLLA and titanium miniplates in 13 and 15 patients, respectively. There were no differences between the two groups in any study variables (e.g., preoperative fracture conditions, postoperative stability during healing, and postoperative complications). Thus, u-HA/PLLA composite plates were presumed to have stability and reliability similar to titanium miniplates when used in endoscopic open reduction and internal fixation of mandibular sub-condylar fractures.

In a 2019 in vitro study, Sukegawa et al. [183] compared u-HA/PLLA and titanium plate systems using the polyurethane hemi-mandible model. They measured tensile and shear strengths, as well as load values in anteroposterior and lateromedial directions, at displacements ranging from 0.5 mm to 5 mm. Because the polyurethane mandible model only replicates the properties of cancellous bone, this constituted a preliminary assessment of stability in the two systems. At displacements of 0.5 mm and 1 mm the titanium fixation system had greater tensile and shear strengths, as well as load values in the anteroposterior direction; however, there were no significant differences between groups in the load values in the anteroposterior direction at displacements of 1.5–5 mm or in the load values in the lateromedial direction at all displacements. Although u-HA/PLLA bioresorbable plates exhibited lower strength, compared to titanium plates, the authors suggested that appropriate placement of the u-HA/PLLA bioresorbable plates could provide adequate strength for the treatment of mandibular sub-condylar fractures. In 2020, a similar study compared biomechanical loading between titanium and u-HA/PLLA screw systems for the fixation of intracapsular condylar fractures [184]. Although this introductory study was conducted using polyurethane replicas of hemi-mandibles, it was the first study concerning the osteosynthesis of mandibular condylar head fractures using the u-HA/PLLA screw system. The study demonstrated that double fixation using either titanium or u-HA/PLLA screws substantially increased the fixation force against loads in both vertical and horizontal directions, while the titanium screws had significantly greater shear resistance and slightly greater resistance to vertical and horizontal loads for small displacements. The authors suggested that intermaxillary fixation should be considered with the application of a single screw because of anatomical complexities. Notably, dietary guidance and elastics are required when u-HA/PLLA screws are used in patients with high occlusal forces. In conclusion, although mechanical strength is lower in the u-HA/PLLA system than in titanium systems, it remains useful for the fixation of mandibular sub-condylar and condylar head fractures because of its biocompatibility and osteoconductivity.

### 3.2. Orthognathic Surgery

For over 30 years, titanium fixation systems have been considered the “gold standard” for rigid fixation in orthognathic surgery because of their superior mechanical strength, biocompatibility, and osseointegration [4,5,6]. Titanium osteofixation systems often require removal because of adverse effects on surrounding tissues [185], radiological interferences, and possible stress-shielding; they may also be removed at the patient’s request. Resorbable devices were developed to eliminate the need for removal surgery. The use of resorbable plates in the field of orthognathic surgery was first reported in 1988 by Haers [186]. Since then, numerous clinical investigations have reported the postoperative skeletal stability and frequency of relapse with the use of resorbable-material fixation in orthognathic surgery [187,188,189,190]. Although some studies have demonstrated good clinical results [186,188,189,190], resorbable osteosynthesis systems (e.g., first-generation and second-generation bioresorbable materials) are not widely used. Matthews [191] noted the lack of segmental stability with resorbable osteosynthesis, particularly in the early postoperative period. In a study concerning PDLLA fixation devices, failure caused by plate breakage in the early postoperative stages was reported in 40 of 685 patients. The earlier bioresorbable materials were radiolucent and difficult to identify on radiographs. The combination of u-HA and PLLA has created a new generation of bioresorbable materials with improved initial strength, bioresorbability, osteoconductivity, and bone-bonding capacity, compared to polymer-only devices. In this section, we evaluate the applications of u-HA/PLLA in orthognathic surgery.

#### 3.2.1. Le Fort I osteotomy

To assess the safety and efficacy of the u-HA/PLLA device system in Le Fort I osteotomies, Ueki et al. conducted multiple studies beginning in 2010. They compared bone healing after Le Fort I osteotomy in 18 Class III patients using PLLA, u-HA/PLLA, and titanium plates [143]. Their results showed that the bone defect volumes were remarkably reduced in three dimensions at 1 year postoperatively, compared to immediately postoperatively; there were no significant differences among plate types [143]. In 2012, they compared maxillary stability after Le Fort I osteotomy in 60 Class III patients using these three types of fixation devices [192]. Although there was a slight difference between u-HA/PLLA and PLLA plating systems, maxillary stability with satisfactory results was achieved in all types of plating systems [192]. They also observed that the bone gaps after Le Fort I osteotomy using these materials were not always completely healed after 1 year [143]. Therefore, they used the self-setting α-tricalcium phosphate (α-TCP; Biopex^®^) to fill the bone defects postoperatively, thus improving bone regeneration and long-term stability. A rabbit model study confirmed that the use of u-HA/PLLA plates with this material was beneficial, i.e., it could provide sufficient bone regeneration while maintaining strength and fixation in the surgical bone defect [193]. A 2013 study evaluated the postoperative changes in maxillary stability after Le Fort I osteotomy using u-HA/PLLA devices with or without α-TCP [194]. It demonstrated that maxillary stability depended on the direction of maxillary movement, rather than the use of α-TCP [194]. Because the maxillary sinus walls are directly influenced by Le Fort I osteotomy, Ueki performed a study to assess the maxillary sinus after Le Fort I osteotomy using PLLA and u-HA/PLLA, with or without α-TCP [195]. There were no significant differences in the sinus area between PLLA and u-HA/PLLA at 1 week and 1 year postoperatively. However, the frequency of intact sinus area was higher in the group with α-TCP than in the group without α-TCP. These findings suggested that the use of this alternative bone material significantly affected sinus conditions; as such, careful use was recommended by the authors [195]. The use of self-setting α-TCP incorporated with resorbable materials in Le Fort I osteotomy did not enhance maxillary stability, although it did support the bone regeneration process.

**Figure 6:**
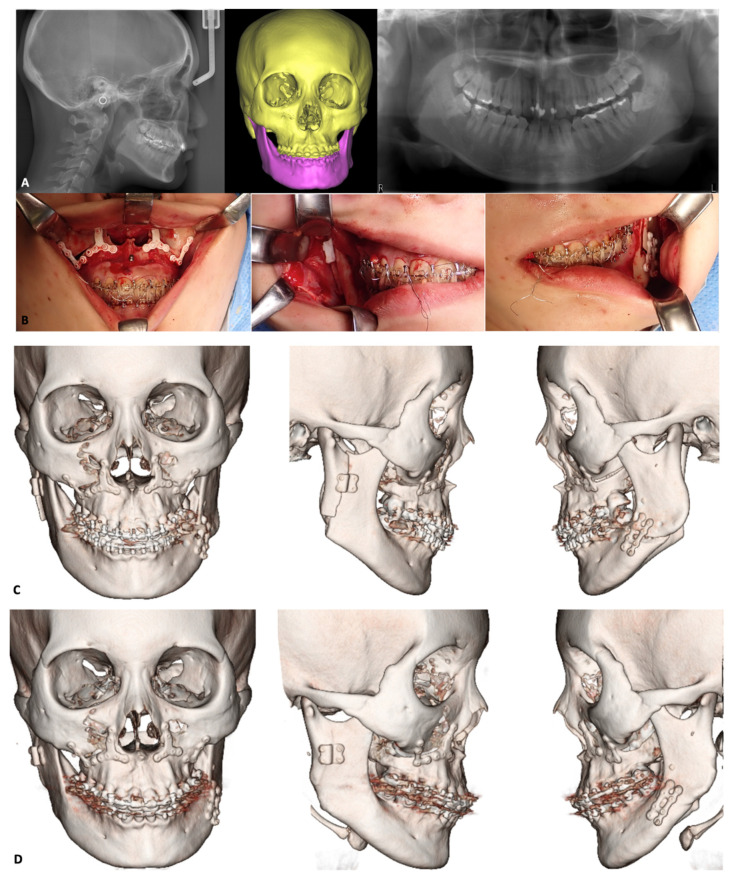
Illustration of orthognathic surgeries using u-HA/PLLA fixation devices. A 23-year-old woman with jaw deformity and facial asymmetry underwent plastic surgery treatment consisting of Le Fort I osteotomy in the maxilla and intraoral vertical ramus osteotomy and split sagittal ramus osteotomy in the mandible (IVSO and SSRO, respectively) using u-HA/PLLA fixation devices. Le Fort I osteotomy was used to correct the maxillary deformity. A 3-mm maxillary impaction was performed on the right side; a 3-mm downward movement with bone grafting was performed on the left side. Two Y-shaped and two L-shaped u-HA/PLLA plates were used for stabilization at the pyriform aperture and zygomatic buttresses, respectively. For the mandible, IVSO was performed to move the right side 6 mm posteriorly, while SSRO was performed to move the left side 10 mm anteriorly. A 4-hole square u-HA/PLLA plate and double u-HA/PLLA straight plates were used to fix bone segments on the right and left sides, respectively. (**A**), Preoperative cephalometric, panoramic, and 3D CT scans showing craniofacial asymmetry caused by abnormal maxillary and mandibular development. (**B**), Intraoperative photographs showing the u-HA/PLLA plates that had been implanted to fix bone segments. (**C**), One-month postoperative 3D CT scans in frontal and lateral views. (**D**), One-year postoperative 3D CT scans showing new bone formation with fading of the folds between bone segments in both jaws despite residual fixation devices.

The results of previous studies confirmed the feasibility of the u-HA/PLLA device system applied in Le Fort I osteotomies. Moreover, the studies demonstrated no complications (e.g., wound infection or dehiscence, bone instability, or long-term malocclusion) during the 1-year follow-up. Therefore, u-HA/PLLA may replace the conventional titanium plating systems as a more optimal choice for Le Fort I osteotomy (Figure 6).

#### 3.2.2. Mandibular Osteotomies

SSRO is commonly used to correct mandibular deformities (e.g., mandibular prognathism or retrognathism) and is often indicated in combination with Le Fort I osteotomy. Although the titanium plating system has excellent mechanical strength for the fixation of bony segments in SSRO, the removal of its titanium plates has been recommended for reasons, including the presence of metal ions [196] and the risk of developing bisphosphonate-related osteonecrosis of the jaw [197]. Because u-HA/PLLA devices have been proven effective in the surgical treatment of mandibular fractures, many researchers have investigated their feasibility in mandibular osteotomies, particularly SSRO (Figure 6).

Since 2011, Ueki et al. have published multiple research papers concerning the applicability of u-HA/PLLA fixation systems in mandibular osteotomies. A study of 60 patients who underwent SSRO using PLLA, u-HA/PLLA, and titanium plating systems revealed that time-course changes in the condylar long-axis and skeletal stability after surgery were comparable among the systems [198]. All patients in that study had been treated with the conventional plate-fixation technique: a miniplate and four monocortical screws placed in the mandibular angle region bilaterally through a transcutaneous approach [199]. Another method based on the miniplates involves placement of two monocortical screws in the distal segment and two bicortical screws in the proximal segment [200]. Nevertheless, most studies using titanium plate systems have concluded that there are no significant differences in postoperative stability among various methods [144]. According to Brasileiro [201], the conditional technique provides a less rigid fixation in SSRO. Therefore, a hybrid technique (i.e., bicortical-monocortical fixation [202]), which combines the conventional technique with an additional bicortical screw placed at the posterior-superior region on each side, was developed to enhance stability and provide rigid fixation after SSRO. Ueki et al. compared hybrid fixation using the u-HA/PLLA system and conventional fixation using the PLLA system. They found that there were no significant differences in terms of temporal changes in condylar long-axis and skeletal stability after surgery; plate breakage within 1 week postoperatively during elastic traction occurred only in the conventional fixation group [203]. In another study of 76 patients who underwent SSRO using monocortical and bicortical plate fixation techniques with the PLLA system or a hybrid fixation technique with the u-HA/PLLA system, Ueki et al. found that postoperative skeletal stability was similar among the three fixation techniques. However, CT scans demonstrated breakage of six resorbable plates with the monocortical plate fixation technique [204]. Based on the results of their two previous studies, Ueki et al. suggested that bicortical and hybrid fixation techniques are reliable methods to improve fixation between bone fragments and prevent the breakage of resorbable plates in SSRO. Although the additional bicortical screws used for strength enhancement may delay the recovery of lower lip hypoesthesia after SSRO, this can be avoided by appropriate positioning [205]. Moreover, Ueki et al. investigated changes in CT values (pixels) of ramus bone and screws after SSRO in patients who had been treated with and without self-setting α-TCP (Biopex^®^). They found that the use of this alternative bone material and the fixation-plate type may affect bone quality during the healing process [206].

Although u-HA/PLLA demonstrated greater mechanical strength, compared to previous resorbable materials, it remains weaker than titanium. Adequate osteosynthesis with minimal morbidity is crucial for the success of orthognathic surgeries that use resorbable plates because rigidity and stability between bony fragments can accelerate bone healing and prevent complications [207]. An in vitro study compared the biomechanical loading values of u-HA/PLLA plating systems in SSRO among three groups: a single u-HA/PLLA straight plate with four screws, double u-HA/PLLA straight plates with eight screws, and a u-HA/PLLA ladder plate with eight screws [208]. The findings indicated that the u-HA/PLLA ladder plate system significantly optimized the resistance and stability of plate fixation. In another clinical study, long-term skeletal stability of the u-HA/PLLA ladder plate system in SSRO was compared with the long-term skeletal stability of titanium miniplates [209]. The anterior displacement of point B at 6 months postoperatively and the inferior displacement at 2 years postoperatively were significantly greater in the titanium group than in the u-HA/PLLA group. This suggested that the use of a u-HA/PLLA ladder plating system in SSRO leads to a stable postoperative mandibular position.

**Figure 7:**
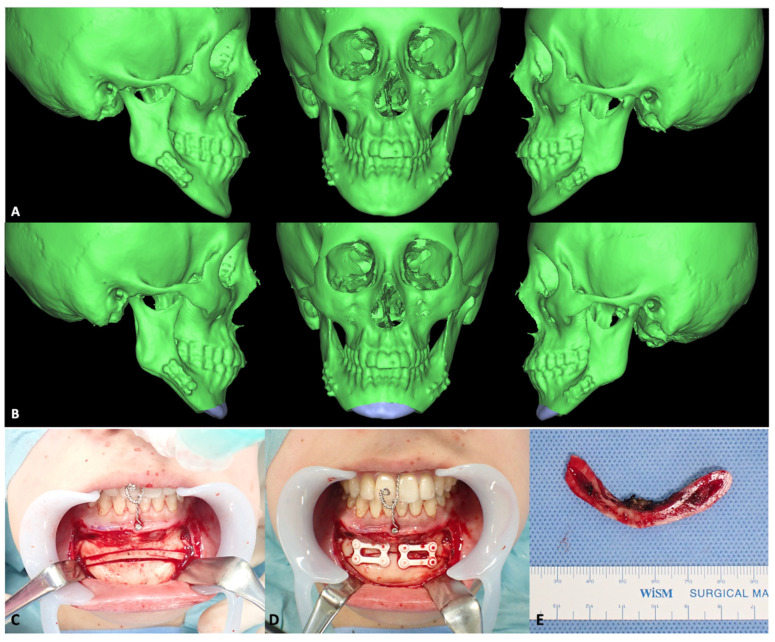

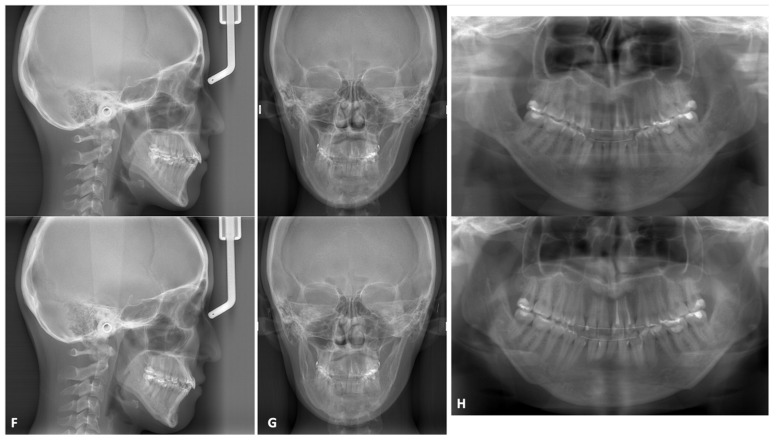
Representative genioplasty images. A 20-year-old woman underwent bilateral SSRO for mandibular protrusion. After 3 years, genioplasty was performed to correct her protruding chin. (**A**), Preoperative 3D CT scans showing anteriorly and inferiorly protruding chin. Notably, this patient had undergone SSRO using u-HA/PLLA plates at that time. (**B**), Chin repositioning was designed using a 3D preoperative simulation program. (**C**), Surgical exposure and osteotomies were performed as planned. (**D**), Fixation of bone segments was performed using two 4-hole u-HA/PLLA rectangle plates. (**E**), Bone fragments were removed from the chin. (**F**–**H**), Cephalometric images, skull anteroposterior views, and panoramic images (respectively) acquired before (**upper**) and after (**lower**) genioplasty. Chin position improved considerably with the operation.

Another common procedure in the mandible is genioplasty (Figure 7). The application of u-HA/PLLA fixation systems in this procedure has attracted the attention of various researchers. Ueki et al. examined chin stability after advancement genioplasty using u-HA/PLLA fixation [145]. In that study, 22 patients were divided into u-HA/PLLA and titanium plate groups. In the u-HA/PLLA plate group, horizontal osteotomy was performed and the central region of the chin was temporarily fixed using a titanium plate and screws; two u-HA/PLLA bicortical screws were then placed bilaterally. The titanium plate and screws were subsequently removed and a u-HA/PLLA plate was implanted to fix the segments. In the titanium plate group, the central region of the chin was fixed rigidly using only a titanium plate and screws. Lateral cephalometric images were acquired before surgery, immediately after surgery, and at 1 year postoperatively. The results showed that the use of a u-HA/PLLA system with initial support from a titanium plate and screws could achieve stability in advancement genioplasty. However, vertical relapse to an inferior position and resorption at the anterosuperior margin of the segment was observed because of forces on the bent part of the plate. Therefore, new prebent u-HA/PLLA plates were developed to solve this problem; these require further investigations to determine their usefulness and stability.

### 3.3. Other Reconstructive Applications

Because of the osteoconductive feature of u-HA/PLLA, it has also been studied for procedures other than fracture and orthognathic surgeries (e.g., sinus lift and alveolar ridge augmentation). Although there are few publications regarding these applications, their results have demonstrated the feasibility, effectiveness, and safety of this material.

**Figure 8:**
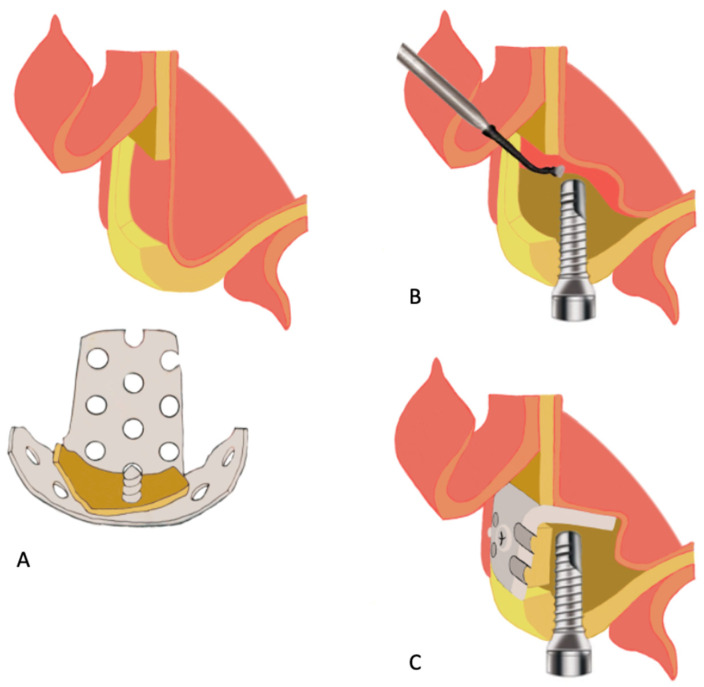
Schematic diagram of nongrafted sinus lift with a u-HA/PLLA mesh plate device based on the technique described by Kaneko [146]. (**A**), Creation of a bone window in the lateral sinus wall using the piezoelectric device and maintenance of the fragmented bone for the final step. (**B**), Cautious elevation of the sinus membrane from the sinus floor, followed by placement of the implant through the maxillary ridge into the space created under the elevated sinus membrane. (**C**), Fixation with a bent u-HA/PLLA plate attached to the fragmented bone using two short u-HA/PLLA screws to cover the bone window.

In 2016, Kaneko et al. reported a case of nongrafted maxillary sinus lift with a u-HA/PLLA device in an atrophic maxilla [146]. A 60-year-old healthy woman with maxillary alveolar ridge atrophy (caused by edentulous maxilla from the right first premolar to the second molar region) underwent a nongrafted sinus lift and simultaneous dental implant placement. After osteotomy of the lateral sinus wall had been performed using a piezoelectric device, a bone window was created and fragmented bone was attached to a u-HA/PLLA plate (Figure 8A). Subsequently, the sinus membrane was carefully elevated from the sinus floor, and two dental implants were placed through the maxillary ridge into the space created under the sinus membrane (Figure 8B). A bent u-HA/PLLA mesh plate attached to the fragmented bone was fixed using two short u-HA/PLLA screws to lift the sinus membrane and cover the bone window (Figure 8C). Bone regeneration around the protruded implants was observed on CT scans at 6 months postoperatively; no intrasinus problems were present, and the abutments were connected. From 6 to 42 months postoperatively, continued vertical bone regeneration was observed in the space under the u-HA/PLLA device. At the end of the 42-month follow-up period, increased bone volume was confirmed above the implant apex and no unnecessary marginal bone loss was evident. These results demonstrated the bioactive and osteoconductive abilities, biocompatibility, and high mechanical strength of u-HA/PLLA material. Notably, UV-treated u-HA/PLLA was superior to untreated u-HA/PLLA in terms of bioactive/osteoconductive abilities for sinus lift in a rabbit model [210]. Other studies have demonstrated enhanced osteoconductivity and biocompatibility of u-HA/PLLA with UV treatment [128,129,130]. These findings may be advantageous for improving the efficacy and safety of this material prior to use in clinical applications.

Recently, the results of alveolar ridge augmentation using u-HA/PLLA screws were investigated in a clinical trial [147]. u-HA/PLLA screws were used to fix the cortical bone block, obtained from the mandibular ramus, to the recipient site. After 6 months, a dental implant was inserted and specimens were harvested using a 2.0-mm trephine bur for further analysis. There were no complications after dental implant placement, and the final prostheses were satisfactory in all patients. A histological assessment revealed that the biomaterial screws were in direct contact with the surrounding bone; there were no inflammatory cells between the bone and the screw. Although fibrous tissue was present around the screw in several specimens, no inflammation or bleeding was observed; these findings confirmed the biocompatibility of u-HA/PLLA. The results of immunohistochemical analysis also supported the bioactive/osteoconductive ability of this material. Overall, the findings suggested that u-HA/PLLA is feasible and safe for application in alveolar ridge augmentation procedures.

## 4. Clinical Complications of u-HA/PLLA

Because of its outstanding properties, including bioactive osteoconductivity, biocompatibility, and high mechanical strengths, u-HA/PLLA biomaterial has achieved great success in maxillofacial surgery. However, some adverse reactions with this bioresorbable material have been reported, presumably in relation to its long degradation time. According to Shikinami et al. [49], the PLLA component was practically absent from the composites after 4.5–5.5 years. In some long-term follow-up studies, u-HA/PLLA was almost completely absorbed by 5 years [172]. Although u-HA/PLLA is highly biocompatible, its prolonged persistence after implantation could lead to adverse reactions, such as inflammation. Numerous studies concerning the applications of u-HA/PLLA have confirmed its safety, but some long-term follow-up investigations have reported notable complications. For example, Hayashi described a patient with subcutaneous swelling in the upper eyelid and histopathological confirmation of inflammatory tissue, 2 years after surgery using u-HA/PLLA plates [172]. Landes also described two patients who exhibited foreign body reactions along with redness and swelling at 15 and 33 months after fracture surgery using u-HA/PLLA devices [140]. Another study described a patient who had been diagnosed with an inflammatory response to a foreign body, based on persistent swelling and redness of the right cheek 2 years after surgical fixation using this material [177]. In 2018, Tatsuta et al. [211] described 13 patients who underwent surgery using the u-HA/PLLA system and required plate removal because of postoperative plate infections. They suggested that the long degradation period had caused foreign body reactions in patients with long-term u-HA/PLLA application.

Another disadvantage of bioresorbable materials is their insufficient mechanical strength. Although u-HA/PLLA showed superior mechanical strength, compared to other materials, it remained inferior to titanium alloys. Hence, this material is limited to applications that do not require high mechanical strength. To address this issue, improvements in surgical techniques and changes in fixation system design have been proposed [205,206,210,211].

Moreover, to match the strength of a titanium plate, the thickness of u-HA/PLLA plates must be increased (1.0–1.4 mm), which may lead to implant palpability and exposure [142]. Fibrous connective tissue may also form, covering the device; this contributes to palpability through thin skin and patient discomfort. Similarly, in the areas of thin mucosae (e.g., para-symphysis), the gingival alveolar portion is fragile and wound dehiscence may occur. Therefore, it is crucial to consider the thickness of the plate and the possible discomfort in areas of thin skin and mucosa, which could increase over time because of fibrous tissue formation around the plate.

## 5. Future Perspectives

To overcome the limitations of u-HA/PLLA composite, a novel bioresorbable composite material has recently been introduced. This material, known as u-HA/PLLA/PGA, combines u-HA particles and a copolymer of PLLA and PGA produced by a forging process, as a fourth-generation bioresorbable biomaterial. Rat models have been used to compare bioactive osteoconductive ability, biocompatibility, and degradation time between u-HA/PLLA/PGA and u-HA/PLLA material. The results showed that bone regeneration ability is comparable between u-HA/PLLA/PGA and u-HA/PLLA, despite a smaller proportion of u-HA (10%) in the new material [71]. Furthermore, immunohistochemical staining revealed CD68-positive cells in a concentrated layer around the u-HA/PLLA and u-HA/PLLA/PGA sheets [72]. Based on the results of these animal studies, the new material has the features of regenerative ability and biocompatibility, and exhibits a shorter resorption time compared to u-HA/PLLA. These studies have initiated a new trend of biodegradable material development for applications in maxillofacial surgery.

## 6. Conclusions

Multiple studies have shown that u-HA/PLLA—a third-generation bioresorbable biomaterial with high mechanical strength, biocompatibility, and bioactive osteoconductivity—is superior to existing bioresorbable materials. Significantly, its bioactive osteoconductivity accelerates postoperative bone healing, producing better results in clinical maxillofacial applications compared to previous bioresorbable materials. However, like other materials, u-HA/PLLA has some limitations, notably, degradation time and mechanical strength. Improved u-HA/PLLA materials that result in new materials with properties suited to particular areas of maxillofacial surgery hold great clinical promise. In the field of oral and maxillofacial reconstructive surgery, the complete replacement of traditional titanium-based systems with bioresorbable materials is inevitable.

## Figures and Tables

**Table 2:** Clinical applications of u-HA/PLLA in oral and maxillofacial surgery.

Maxillofacial Trauma	Orthognathics Surgery	Other Reconstructive Applications
Orbital wall fractures [139] Midfacial fractures - Maxillary fractures [140] - Zygomatic fractures [141] Mandibular fractures [142]	Le Fort I osteotomy [143] Mandibular osteotomies - Sagittal split ramus osteotomy (SSRO) [144] - Genioplasty [145]	Nongrafted maxillary sinus lift [146] Alveolar ridge augmentation [147]

## Data Availability

All data have been illustrated in the manuscript.

## References

[1] Montovani J.C., de Campos L.M.P., Gomes M.A., de Moraes V.R.S., Ferreira F.D., Nogueira E.A. (2006). Etiology and incidence facial fractures in children and adults. Braz. J. Otorhinolaryngol. Engl. Ed..

[2] Gruss J.S. (1990). Complex Craniomaxillofacial Trauma: Evolving Concepts in Management. A Trauma Unit’s Experience—1989 Fraser B. Gurd Lecture. J. Trauma Acute Care Surg..

[3] Bell R., Kiyak H.A., Joondeph D.R., McNeill R.W., Wallen T.R. (1985). Perceptions of facial profile and their influence on the decision to undergo orthognathic surgery. Am. J. Orthod..

[4] Guo S., Qu X., He X., Zhou T., Duan B. (2006). Powder injection molding of Ti–6Al–4V alloy. J. Mater. Process. Technol..

[5] Quinn R.K., Armstrong N.R. (1978). Electrochemical and Surface Analytical Characterization of Titanium and Titanium Hydride Thin Film Electrode Oxidation. J. Electrochem. Soc..

[6] Leventhal G.S. (1951). Titanium, a metal for surgery. J. Bone Joint Surg. Am..

[7] Francel T.J., Birely B.C., Ringelman P.R., Manson P.N. (1992). The fate of plates and screws after facial fracture reconstruction. Plast. Reconstr. Surg..

[8] Kahnberg K.E., Engström H. (1987). Recovery of maxillary sinus and tooth sensibility after le Fort I osteotomy. Br. J. Oral Maxillofac. Surg..

[9] Resnick J.I., Kinney B.M., Kawamoto H.K. (1990). The effect of rigid internal fixation on cranial growth. Ann. Plast. Surg..

[10] Lin K.Y., Bartlett S.P., Yaremchuk M.J., Grossman R.F., Udupa J.K., Whitaker L.A. (1991). An experimental study on the effect of rigid fixation on the developing craniofacial skeleton. Plast. Reconstr. Surg..

[11] Eppley B.L., Platis J.M., Sadove A.M. (1993). Experimental effects of bone plating in infancy on craniomaxillofacial skeletal growth. Cleft Palate-Craniofacial J..

[12] Gilardino M.S., Chen E., Bartlett S.P. (2009). Choice of Internal Rigid Fixation Materials in the Treatment of Facial Fractures. Craniomaxillofacial Trauma Reconstr..

[13] Pan Z., Patil P.M. (2014). Titanium osteosynthesis hardware in maxillofacial trauma surgery: To remove or remain? A retrospective study. Eur. J. Trauma Emerg. Surg. Off. Publ. Eur. Trauma Soc..

[14] Haers P.E., Suuronen R., Lindqvist C., Sailer H. (1998). Biodegradable polylactide plates and screws in orthognathic surgery: Technical note. J. Cranio-Maxillofacial Surg..

[15] Buijs G.J., van Bakelen N.B., Jansma J., de Visscher J.G.A.M., Hoppenreijs T.J.M., Bergsma J.E., Stegenga B., Bos R.R.M. (2012). A randomized clinical trial of biodegradable and titanium fixation systems in maxillofacial surgery. J. Dent. Res..

[16] van Bakelen N.B., Buijs G.J., Jansma J., de Visscher J.G.A.M., Hoppenreijs T.J.M., Bergsma J.E., Stegenga B., Bos R.R.M. (2014). Decision-making considerations in application of biodegradable fixation systems in maxillofacial surgery—A retrospective cohort study. J. Cranio-Maxillofacial Surg..

[17] Kulkarni R.K., Pani K.C., Neuman C., Leonard F. (1966). Polylactic acid for surgical implants. Arch. Surg. Chic..

[18] Cutright D.E., Hunsuck E.E., Beasley J.D. (1971). Fracture reduction using a biodegradable material, polylactic acid. J. Oral Surg. Am. Dent. Assoc..

[19] Shikinami Y., Okuno M. (1999). Bioresorbable devices made of forged composites of hydroxyapatite (HA) particles and poly-L-lactide (PLLA): Part I. Basic characteristics. Biomaterials.

[20] Piskin E. (1995). Biodegradable polymers as biomaterials. J. Biomater. Sci. Polym. Ed..

[21] Yoda R. (1998). Elastomers for biomedical applications. J. Biomater. Sci. Polym. Ed..

[22] Saad B., Neuenschwander P., Uhlschmid G.K., Suter U.W. (1999). New versatile, elastomeric, degradable polymeric materials for medicine. Int. J. Biol. Macromol..

[23] Chu C. (2003). Biodegradable polymeric biomaterials: An updated overview. Biomaterials.

[24] Suuronen R., Kallela I., Lindqvist C. (2000). Bioabsorbable plates and screws: Current state of the art in facial fracture repair. J. Cranio-Maxillofac. Trauma.

[25] Mohamed-Hashem I.K., Mitchell D.A. (2000). Resorbable implants (plates and screws) in orthognathic surgery. J. Orthod..

[26] Ylikontiola L., Sundqvuist K., Sàndor G.K.B., Törmälä P., Ashammakhi N. (2004). Self-reinforced bioresorbable poly-L/DL-lactide [SR-P(L/DL)LA] 70/30 miniplates and miniscrews are reliable for fixation of anterior mandibular fractures: A pilot study. Oral Surg. Oral Med. Oral Pathol. Oral Radiol. Endod..

[27] Edwards R.C., Kiely K.D., Eppley B.L. (2001). The fate of resorbable poly-L-lactic/polyglycolic acid (LactoSorb) bone fixation devices in orthognathic surgery. J. Oral Maxillofac. Surg..

[28] Peltoniemi H.H., Tulamo R.M., Toivonen T., Hallikainen D., Törmälä P., Waris T. (1999). Biodegradable semirigid plate and miniscrew fixation compared with rigid titanium fixation in experimental calvarial osteotomy. J. Neurosurg..

[29] Törmälä P. (1992). Biodegradable self-reinforced composite materials; Manufacturing structure and mechanical properties. Clin. Mater..

[30] Bell R.B., Kindsfater C.S. (2006). The Use of Biodegradable Plates and Screws to Stabilize Facial Fractures. J. Oral Maxillofac. Surg..

[31] Seppänen-Kaijansinkko R., Lindqvist C. (2019). Bioresorbable Materials for Bone Fixation: Review of Biological Concepts and Mechanical Aspects.

[32] Gajjar C., King M. (2014). Resorbable Fiber-Forming Polymers for Biotextile Applications.

[33] Lopes M.S., Jardini A.L., Filho R.M. (2012). Poly (Lactic Acid) Production for Tissue Engineering Applications. Procedia Eng..

[34] Pihlajamaki H. (1992). Absorbable pins of self-reinforced poly-L-lactc acid for fixation of fractures and osteotomies. J Bone Jt. Surg..

[35] Luckachan G.E., Pillai C.K.S. (2011). Biodegradable Polymers- A Review on Recent Trends and Emerging Perspectives. J. Polym. Environ..

[36] Kricheldorf H.R. (2001). Syntheses and application of polylactides. Chemosphere.

[37] Middleton J.C., Tipton A.J. (2000). Synthetic biodegradable polymers as orthopedic devices. Biomaterials.

[38] Bergsma J.E., Rozema F.R., Bos R.R., Boering G., de Bruijn W.C., Pennings A.J. (1995). In vivo degradation and biocompatibility study of in vitro pre-degraded as-polymerized polyactide particles. Biomaterials.

[39] Park Y.-W. (2015). Bioabsorbable osteofixation for orthognathic surgery. Maxillofac. Plast. Reconstr. Surg..

[40] Pina S., Ferreira J. (2012). Bioresorbable Plates and Screws for Clinical Applications: A Review. J. Health Eng..

[41] Mazzonetto R., Paza A.O., Spagnoli D.B. (2004). A retrospective evaluation of rigid fixation in orthognathic surgery using a biodegradable self-reinforced (70L:30DL) polylactide. Int. J. Oral Maxillofac. Surg..

[42] Kim B.C., Padwa B.L., Park H.-S., Jung Y.-S. (2011). Stability of Maxillary Position After Le Fort I Osteotomy Using Self-Reinforced Biodegradable Poly-70L/30DL-Lactide Miniplates and Screws. J. Oral Maxillofac. Surg..

[43] Miller R.A., Brady J.M., Cutright D.E. (1977). Degradation rates of oral resorbable implants (polylactates and polyglycolates): Rate modification with changes in PLA/PGA copolymer ratios. J. Biomed. Mater. Res..

[44] Wiltfang J., Merten H.A., Schultze-Mosgau S., Schrell U., Wénzel D., Kessler P. (2000). Biodegradable miniplates (LactoSorb): Long-term results in infant minipigs and clinical results. J. Craniofac. Surg..

[45] Eppley B.L., Prevel C.D., Sadove A.M., Sarver D. (1996). Resorbable bone fixation: Its potential role in cranio-maxillofacial trauma. J. Cranio-Maxillofac. Trauma.

[46] Eppley B.L., Reilly M. (1997). Degradation characteristics of PLLA-PGA bone fixation devices. J. Craniofac. Surg..

[47] Quereshy F.A., Goldstein J.A., Goldberg J.S., Beg Z. (2000). The efficacy of bioresorbable fixation in the repair of mandibular fractures: An animal study. J. Oral Maxillofac. Surg..

[48] Sukegawa S., Kanno T., Matsumoto K., Sukegawa-Takahashi Y., Masui M., Furuki Y. (2018). Complications of a poly-L-lactic acid and polyglycolic acid osteosynthesis device for internal fixation in maxillofacial surgery. Odontology.

[49] Shikinami Y., Matsusue Y., Nakamura T. (2005). The complete process of bioresorption and bone replacement using devices made of forged composites of raw hydroxyapatite particles/poly l-lactide (F-u-HA/PLLA). Biomaterials.

[50] Barth J., Akritopoulos P., Graveleau N., Barthelemy R., Toanen C., Saffarini M. (2016). Efficacy of Osteoconductive Ceramics in Bioresorbable Screws for Anterior Cruciate Ligament Reconstruction: A Prospective Intrapatient Comparative Study. Orthop. J. Sports Med..

[51] Dong Q.N., Kanno T., Bai Y., Sha J., Hideshima K. (2019). Bone Regeneration Potential of Uncalcined and Unsintered Hydroxyapatite/Poly l-lactide Bioactive/Osteoconductive Sheet Used for Maxillofacial Reconstructive Surgery: An In Vivo Study. Materials.

[52] Matsumoto M., Chosa E., Nabeshima K., Shikinami Y., Tajima N. (2002). Influence of bioresorbable, unsintered hydroxyapatite/poly-L-lactide composite films on spinal cord, nerve roots, and epidural space. J. Biomed. Mater. Res..

[53] Wypych G., Wypych G. (2018). 6—Functional Fillers—Applications. Functional Fillers.

[54] Hasegawa S., Tamura J., Neo M., Goto K., Shikinami Y., Saito M., Nakamura T. (2005). In Vivo Evaluation of Porous Hydroxyapatite/Poly D/L-Lactide Composite for Bone Substitute and Scaffold. Key Eng. Mater..

[55] Hasegawa S., Tamura J., Neo M., Fujibayashi S., Goto K., Shikinami Y., Okazaki K., Nakamura T. (2006). In Vivo Evaluation of Porous Hydroxyapatite/Poly D/L-lactide Composite for Bone Substitutes and Scaffolds. Key Eng. Mater..

[56] Bai Y., Kanno T., Tatsumi H., Miyamoto K., Sha J., Hideshima K., Matsuzaki Y. (2018). Feasibility of a Three-Dimensional Porous Uncalcined and Unsintered Hydroxyapatite/poly-d/l-lactide Composite as a Regenerative Biomaterial in Maxillofacial Surgery. Materials.

[57] Akino N., Tachikawa N., Munakata M., Kasugai S. (2013). The Use of Porous Composite Uncalcined Hydroxyapatite / poly-DL-lactide for Vertical Ridge Augmentation. J. Oral Tissue Eng..

[58] Akino N., Tachikawa N., Miyahara T., Ikumi R., Kasugai S. (2019). Vertical ridge augmentation using a porous composite of uncalcined hydroxyapatite and poly-DL-lactide enriched with types 1 and 3 collagen. Int. J. Implant Dent..

[59] Manoukian O., Sardashti N., Stedman T., Gailiunas K., Ojha A., Penalosa A., Mancuso C., Hobert M., Kumbar S. (2018). Biomaterials for Tissue Engineering and Regenerative Medicine.

[60] Schumann P., Lindhorst D., Wagner M.E.H., Schramm A., Gellrich N.-C., Rücker M. (2013). Perspectives on resorbable osteosynthesis materials in craniomaxillofacial surgery. Pathobiol. J. Immunopathol. Mol. Cell. Biol..

[61] Göktürk E., Erdal H. (2017). Biomedical Applications of Polyglycolic Acid (PGA). Sak. Üniversitesi Fen Bilim. Enstitüsü Derg..

[62] Chan C.M., Vandi L.-J., Pratt S., Halley P., Richardson D., Werker A., Laycock B. (2018). Composites of Wood and Biodegradable Thermoplastics: A Review. Polym. Rev..

[63] Farah S., Anderson D.G., Langer R. (2016). Physical and mechanical properties of PLA, and their functions in widespread applications—A comprehensive review. Adv. Drug Deliv. Rev..

[64] Ramot Y., Haim-Zada M., Domb A.J., Nyska A. (2016). Biocompatibility and safety of PLA and its copolymers. Adv. Drug Deliv. Rev..

[65] Bergsma J.E., de Bruijn W.C., Rozema F.R., Bos R.R., Boering G. (1995). Late degradation tissue response to poly(L-lactide) bone plates and screws. Biomaterials.

[66] DeStefano V., Khan S., Tabada A. (2020). Applications of PLA in modern medicine. Eng. Regen..

[67] Rasool F., Ahmad M., Khan H.M., Khan S.A., Murtaza G. (2011). Comparative dissolution study of metoprolol tartrate loaded PLGA (50: 50) and PLGA (75: 25) microparticles. Asian J. Chem..

[68] Gentile P., Chiono V., Carmagnola I., Hatton P.V. (2014). An Overview of Poly(lactic-co-glycolic) Acid (PLGA)-Based Biomaterials for Bone Tissue Engineering. Int. J. Mol. Sci..

[69] Tanaka K., Takemoto M., Fujibayashi S., Neo M., Shikinami Y., Nakamura T. (2011). A bioactive and bioresorbable porous cubic composite scaffold loaded with bone marrow aspirate: A potential alternative to autogenous bone grafting. Spine.

[70] Kanno T., Sukegawa S., Furuki Y., Nariai Y., Sekine J. (2018). Overview of innovative advances in bioresorbable plate systems for oral and maxillofacial surgery. Jpn. Dent. Sci. Rev..

[71] Ngo H.X., Dong Q.N., Bai Y., Sha J., Ishizuka S., Okui T., Sukegawa S., Kanno T. (2020). Bone Regeneration Capacity of Newly Developed Uncalcined/Unsintered Hydroxyapatite and Poly-l-lactide-co-glycolide Sheet in Maxillofacial Surgery: An In Vivo Study. Nanomaterials.

[72] Ishizuka S., Dong Q.N., Ngo H.X., Bai Y., Sha J., Toda E., Okui T., Kanno T. (2021). Bioactive Regeneration Potential of the Newly Developed Uncalcined/Unsintered Hydroxyapatite and Poly-l-Lactide-Co-Glycolide Biomaterial in Maxillofacial Reconstructive Surgery: An In Vivo Preliminary Study. Materials.

[73] Kawai H., Sukegawa S., Nakano K., Takabatake K., Ono S., Nagatsuka H., Furuki Y. (2021). Biological Effects of Bioresorbable Materials in Alveolar Ridge Augmentation: Comparison of Early and Slow Resorbing Osteosynthesis Materials. Materials.

[74] Batchelar D.L., Davidson M.T.M., Dabrowski W., Cunningham I.A. (2006). Bone-composition imaging using coherent-scatter computed tomography: Assessing bone health beyond bone mineral density. Med. Phys..

[75] Malmberg P., Nygren H. (2008). Methods for the analysis of the composition of bone tissue, with a focus on imaging mass spectrometry (TOF-SIMS). Proteomics.

[76] Hu Y.-Y., Rawal A., Schmidt-Rohr K. (2010). Strongly bound citrate stabilizes the apatite nanocrystals in bone. Proc. Natl. Acad. Sci.USA.

[77] Märten A., Fratzl P., Paris O., Zaslansky P. (2010). On the mineral in collagen of human crown dentine. Biomaterials.

[78] Vallet-Regí M., González-Calbet J.M. (2004). Calcium phosphates as substitution of bone tissues. Prog. Solid State Chem..

[79] Hench L.L. (1998). Bioceramics. J. Am. Ceram. Soc..

[80] Xiao W., Sonny Bal B., Rahaman M.N. (2016). Preparation of resorbable carbonate-substituted hollow hydroxyapatite microspheres and their evaluation in osseous defects in vivo. Mater. Sci. Eng. C Mater. Biol. Appl..

[81] Sadat-Shojai M., Khorasani M.-T., Dinpanah-Khoshdargi E., Jamshidi A. (2013). Synthesis methods for nanosized hydroxyapatite with diverse structures. Acta Biomater..

[82] Gomes D.S., Santos A.M.C., Neves G.A., Menezes R.R., Gomes D.S., Santos A.M.C., Neves G.A., Menezes R.R. (2019). A brief review on hydroxyapatite production and use in biomedicine. Cerâmica.

[83] Cai Y., Liu Y., Yan W., Hu Q., Tao J., Zhang M., Shi Z., Tang R. (2007). Role of hydroxyapatite nanoparticle size in bone cell proliferation. J. Mater. Chem..

[84] Li B., Guo B., Fan H., Zhang X. (2008). Preparation of nano-hydroxyapatite particles with different morphology and their response to highly malignant melanoma cells in vitro. Appl. Surf. Sci..

[85] Xu J.L., Khor K.A., Sui J.J., Zhang J.H., Chen W.N. (2009). Protein expression profiles in osteoblasts in response to differentially shaped hydroxyapatite nanoparticles. Biomaterials.

[86] Wang A., Lu Y., Zhu R.-F., Li S., Ma X. (2009). Effect of process parameters on the performance of spray dried hydroxyapatite microspheres. Powder Technol..

[87] Bai Y., Sha J., Kanno T., Miyamoto K., Hideshima K., Matsuzaki Y. (2021). Comparison of the Bone Regenerative Capacity of Three-Dimensional Uncalcined and Unsintered Hydroxyapatite/Poly-d/l-Lactide and Beta-Tricalcium Phosphate Used as Bone Graft Substitutes. J. Investig. Surg. Off. J. Acad. Surg. Res..

[88] LeGeros: Calcium Phosphate Biomaterials: Preparation,...—Google Scholar. https://scholar.google.com/scholar_lookup?title=Calcium%20phosphate%20biomaterials%3A%20preparation%2C%20properties%20and%20biodegradation&author=R.Z..%20LeGeros&author=J.P..%20LeGeros&author=G..%20Daculsi&author=R..%20Kijkowska&pages=1429&publication_year=1995.

[89] Ducheyne P., Radin S., King L. (1993). The effect of calcium phosphate ceramic composition and structure on in vitro behavior. I. Dissolution. J. Biomed. Mater. Res..

[90] Ducheyne P., Qiu Q. (1999). Bioactive ceramics: The effect of surface reactivity on bone formation and bone cell function. Biomaterials.

[91] Porter A.E., Hobbs L.W., Rosen V.B., Spector M. (2002). The ultrastructure of the plasma-sprayed hydroxyapatite–bone interface predisposing to bone bonding. Biomaterials.

[92] Bertazzo S., Zambuzzi W.F., Campos D.D.P., Ogeda T.L., Ferreira C.V., Bertran C.A. (2010). Hydroxyapatite surface solubility and effect on cell adhesion. Colloids Surf. B Biointerfaces.

[93] Grandfield K., Palmquist A., Engqvist H., Thomsen P. (2012). Resolving the CaP-bone interface. Biomatter.

[94] Wang K., Zhou C., Hong Y., Zhang X. (2012). A review of protein adsorption on bioceramics. Interface Focus.

[95] Koike T., Sha J., Bai Y., Matsuda Y., Hideshima K., Yamada T., Kanno T. (2019). Efficacy of Bacterial Cellulose as a Carrier of BMP-2 for Bone Regeneration in a Rabbit Frontal Sinus Model. Materials.

[96] Webster T.J., Siegel R.W., Bizios R. (1999). Osteoblast adhesion on nanophase ceramics. Biomaterials.

[97] Webster T.J., Schadler L.S., Siegel R.W., Bizios R. (2001). Mechanisms of enhanced osteoblast adhesion on nanophase alumina involve vitronectin. Tissue Eng..

[98] Webster T.J., Ahn E.S. (2007). Nanostructured biomaterials for tissue engineering bone. Adv. Biochem. Eng. Biotechnol..

[99] Liu H., Webster T.J. (2007). Nanomedicine for implants: A review of studies and necessary experimental tools. Biomaterials.

[100] Duan Y.-R., Wang C., Chen J., Zhang X. (2004). A Study of Bone-Like Apatite Formation on Calcium Phosphate Ceramics in Different Simulated Body Fluids (SBF). Key Eng. Mater..

[101] Duan Y.-R., Zhang Z.-R., Wang C., Chen J., Zhang X. (2004). Apatite Formation on HA/TCP Ceramics in Dynamic Simulated Body Fluid. Key Eng. Mater..

[102] Li X., van Blitterswijk C.A., Feng Q., Cui F., Watari F. (2008). The effect of calcium phosphate microstructure on bone-related cells in vitro. Biomaterials.

[103] Sha J., Kanno T., Miyamoto K., Bai Y., Hideshima K., Matsuzaki Y. (2019). Application of a Bioactive/Bioresorbable Three-Dimensional Porous Uncalcined and Unsintered Hydroxyapatite/Poly-d/l-lactide Composite with Human Mesenchymal Stem Cells for Bone Regeneration in Maxillofacial Surgery: A Pilot Animal Study. Materials.

[104] Shimazaki K., Mooney V. (1985). Comparative study of porous hydroxyapatite and tricalcium phosphate as bone substitute. J. Orthop. Res. Off. Public Orthop. Res. Soc..

[105] van Blitterswijk C.A., Grote J.J., Kuÿpers W., Blok-van Hoek C.J., Daems W.T. (1985). Bioreactions at the tissue/hydroxyapatite interface. Biomaterials.

[106] van Blitterswijk C.A., Grote J.J., Kuijpers W., Daems W.T., de Groot K. (1986). Macropore tissue ingrowth: A quantitative and qualitative study on hydroxyapatite ceramic. Biomaterials.

[107] Winter M., Griss P., de Groot K., Tagai H., Heimke G., von Dijk H.J., Sawai K. (1981). Comparative histocompatibility testing of seven calcium phosphate ceramics. Biomaterials.

[108] Toda E., Bai Y., Sha J., Dong Q.N., Ngo H.X., Suyama T., Miyamoto K., Matsuzaki Y., Kanno T. (2021). Feasibility of Application of the Newly Developed Nano-Biomaterial, β-TCP/PDLLA, in Maxillofacial Reconstructive Surgery: A Pilot Rat Study. Nanomaterials.

[109] Inoue O., Ibaraki K., Shimabukuro H., Shingaki Y. (1993). Packing with high-porosity hydroxyapatite cubes alone for the treatment of simple bone cyst. Clin. Orthop..

[110] Schepers E., de Clercq M., Ducheyne P., Kempeneers R. (1991). Bioactive glass particulate material as a filler for bone lesions. J. Oral Rehabil..

[111] Goto T., Kojima T., Iijima T., Yokokura S., Kawano H., Yamamoto A., Matsuda K. (2001). Resorption of synthetic porous hydroxyapatite and replacement by newly formed bone. J. Orthop. Sci..

[112] Silverstein S.C., Steinman R.M., Cohn Z.A. (1977). Endocytosis. Annu. Rev. Biochem..

[113] Oonishi H., Hench L.L., Wilson J., Sugihara F., Tsuji E., Kushitani S., Iwaki H. (1999). Comparative bone growth behavior in granules of bioceramic materials of various sizes. J. Biomed. Mater. Res..

[114] Masutani K., Kimura Y. (2014). Chapter 1 PLA Synthesis. From the Monomer to the Polymer.

[115] Esen A., Ataoğlu H., Gemi L. (2008). Comparison of stability of titanium and absorbable plate and screw fixation for mandibular angle fractures. Oral Surg. Oral Med. Oral Pathol. Oral Radiol. Endod..

[116] Bayram B., Araz K., Uckan S., Balcik C. (2009). Comparison of fixation stability of resorbable versus titanium plate and screws in mandibular angle fractures. J. Oral Maxillofac. Surg..

[117] Sukegawa S., Kanno T., Nagano D., Shibata A., Sukegawa-Takahashi Y., Furuki Y. (2016). The Clinical Feasibility of Newly Developed Thin Flat-Type Bioresorbable Osteosynthesis Devices for the Internal Fixation of Zygomatic Fractures: Is There a Difference in Healing Between Bioresorbable Materials and Titanium Osteosynthesis?. J. Craniofac. Surg..

[118] Bostman O. (1991). Current concepts review; Absorbable implants for the fixation of fractures. J Bone Jt. Surg. A.

[119] Böstman O.M. (1998). Osteoarthritis of the ankle after foreign-body reaction to absorbable pins and screws: A three- to nine-year follow-up study. J. Bone Joint Surg. Br..

[120] Tegnander A., Engebretsen L., Bergh K., Eide E., Holen K., Iversen O.-J. (1994). Activation of the complement system and adverse effects of biodegradable pins of poly-lactic acid (Biofix^®^) in osteochondritis dissecans. Acta Orthop..

[121] Bergsma E.J., Rozema F.R., Bos R.R.M., Bruijn W.C.D. (1993). Foreign body reactions to resorbable poly(l-lactide) bone plates and screws used for the fixation of unstable zygomatic fractures. J. Oral Maxillofac. Surg..

[122] Matsusue Y., Nakamura T., Iida H., Shimizu K. (1997). A long-term clinical study on drawn poly-L-lactide implants in orthopaedic surgery. J. Long. Term Eff. Med. Implant.

[123] Matsusue Y., Nakamura T., Suzuki S., Iwasaki R. (1996). Biodegradable pin fixation of osteochondral fragments of the knee. Clin. Orthop..

[124] Ahl T., Dalén N., Lundberg A., Wykman A. (1994). Biodegradable fixation of ankle fractures. A roentgen stereophotogrammetric study of 32 cases. Acta Orthop. Scand..

[125] Donigian A.M., Plaga B.R., Caskey P.M. (1993). Biodegradable fixation of physeal fractures in goat distal femur. J. Pediatr. Orthop..

[126] Böstman O., Hirvensalo E., Vainionpää S., Mäkelä A., Vihtonen K., Törmälä P., Rokkanen P. (1989). Ankle fractures treated using biodegradable internal fixation. Clin. Orthop..

[127] Yasunaga T., Matsusue Y., Furukawa T., Shikinami Y., Okuno M., Nakamura T. (1999). Bonding behavior of ultrahigh strength unsintered hydroxyapatite particles/poly(L-lactide) composites to surface of tibial cortex in rabbits. J. Biomed. Mater. Res..

[128] Ikumi R., Miyahara T., Akino N., Tachikawa N., Kasugai S. (2018). Guided bone regeneration using a hydrophilic membrane made of unsintered hydroxyapatite and poly(L-lactic acid) in a rat bone-defect model. Dent. Mater. J..

[129] Ikawa H., Moroi A., Yoshizawa K., Saida Y., Hotta A., Tsutsui T., Fukaya K., Hiraide R., Takayama A., Tsunoda T. (2017). Bone regeneration enhancement by ultra-violet (UV) treatment for uHA/PLLA absorbable mesh. J. Cranio-Maxillofacial Surg..

[130] Moroi A., Okuno M., Kobayashi G., Gamo H., Serizawa I., Yoshizawa K., Ikawa H., Ueki K. (2016). Effect on surface character and mechanical property of unsintered hydroxyapatite/poly-l-lactic acid (uHA/PLLA) material by UV treatment. J. Biomed. Mater. Res. B Appl. Biomater..

[131] Furukawa T., Matsusue Y., Yasunaga T., Shikinami Y., Okuno M., Nakamura T. (2000). Biodegradation behavior of ultra-high-strength hydroxyapatite/poly (l-lactide) composite rods for internal fixation of bone fractures. Biomaterials.

[132] Ishii S., Tamura J., Furukawa T., Nakamura T., Matsusue Y., Shikinami Y., Okuno M. (2003). Long-term study of high-strength hydroxyapatite/poly(L-lactide) composite rods for the internal fixation of bone fractures: A 2-4-year follow-up study in rabbits. J. Biomed. Mater. Res. B Appl. Biomater..

[133] Hasegawa S., Ishii S., Tamura J., Furukawa T., Neo M., Matsusue Y., Shikinami Y., Okuno M., Nakamura T. (2006). A 5-7 year in vivo study of high-strength hydroxyapatite/poly(L-lactide) composite rods for the internal fixation of bone fractures. Biomaterials.

[134] Törmälä P., Vasenius J., Vainionpää S., Laiho J., Pohjonen T., Rokkanen P. (1991). Ultra-high-strength absorbable self-reinforced polyglycolide (SR-PGA) composite rods for internal fixation of bone fractures: In vitro and in vivo study. J. Biomed. Mater. Res..

[135] Majola A., Vainionpää S., Rokkanen P., Mikkola H.-M., Törmälä P. (1992). Absorbable self-reinforced polylactide (SR-PLA) composite rods for fracture fixation: Strength and strength retention in the bone and subcutaneous tissue of rabbits. J. Mater. Sci. Mater. Med..

[136] Matsusue Y., Yamamuro T., Oka M., Shikinami Y., Hyon S.H., Ikada Y. (1992). In vitro and in vivo studies on bioabsorbable ultra-high-strength poly(L-lactide) rods. J. Biomed. Mater. Res..

[137] Hiatt J.L. (2009). Textbook of Head and Neck Anatomy.

[138] Breeland G., Aktar A., Patel B.C. (2021). Anatomy, Head and Neck, Mandible. StatPearls.

[139] Park H., Kim H.-S., Lee B.-I. (2015). Medial Wall Orbital Reconstruction using Unsintered Hydroxyapatite Particles/Poly L-Lactide Composite Implants. Arch. Craniofacial Surg..

[140] Landes C., Ballon A., Ghanaati S., Tran A., Sader R. (2014). Treatment of malar and midfacial fractures with osteoconductive forged unsintered hydroxyapatite and poly-L-lactide composite internal fixation devices. J. Oral Maxillofac. Surg..

[141] Kim S.Y., Nam S.M., Park E.S., Kim Y.B. (2019). Evaluation of one-point fixation for zygomaticomaxillary complex fractures using a three-dimensional photogrammetric analysis. J. Otolaryngol.-Head Neck Surg..

[142] Sukegawa S., Kanno T., Katase N., Shibata A., Takahashi Y., Furuki Y. (2016). Clinical Evaluation of an Unsintered Hydroxyapatite/Poly-L-Lactide Osteoconductive Composite Device for the Internal Fixation of Maxillofacial Fractures. J. Craniofac. Surg..

[143] Ueki K., Miyazaki M., Okabe K., Mukozawa A., Marukawa K., Moroi A., Nakagawa K., Yamamoto E. (2011). Assessment of bone healing after Le Fort I osteotomy with 3-dimensional computed tomography. J. Cranio-Maxillofacial Surg..

[144] Ueki K., Hashiba Y., Marukawa K., Alam S., Nakagawa K., Yamamoto E. (2008). Skeletal Stability After Mandibular Setback Surgery: Bicortical Fixation Using a 2.0-mm Locking Plate System Versus Monocortical Fixation Using a Nonlocking Plate System. J. Oral Maxillofac. Surg..

[145] Ueki K., Moroi A., Yoshizawa K. (2019). Stability of the chin after advancement genioplasty using absorbable plate and screws with template devices. J. Cranio-Maxillofacial Surg..

[146] Kaneko T., Nakamura S., Hino S., Horie N., Shimoyama T. (2016). Continuous intra-sinus bone regeneration after nongrafted sinus lift with a PLLA mesh plate device and dental implant placement in an atrophic posterior maxilla: A case report. Int. J. Implant Dent..

[147] Sukegawa S., Kawai H., Nakano K., Takabatake K., Kanno T., Nagatsuka H., Furuki Y. (2019). Advantage of Alveolar Ridge Augmentation with Bioactive/Bioresorbable Screws Made of Composites of Unsintered Hydroxyapatite and Poly-L-lactide. Materials.

[148] Dubois L., Steenen S.A., Gooris P.J.J., Bos R.R.M., Becking A.G. (2016). Controversies in orbital reconstruction-III. Biomaterials for orbital reconstruction: A review with clinical recommendations. Int. J. Oral Maxillofac. Surg..

[149] Ilankovan V., Jackson I.T. (1992). Experience in the use of calvarial bone grafts in orbital reconstruction. Br. J. Oral Maxillofac. Surg..

[150] Chowdhury K., Krause G.E. (1998). Selection of materials for orbital floor reconstruction. Arch. Otolaryngol. Head Neck Surg..

[151] Peng M.Y., Merbs S.L., Grant M.P., Mahoney N.R. (2017). Orbital fracture repair outcomes with preformed titanium mesh implants and comparison to porous polyethylene coated titanium sheets. J. Cranio-Maxillofacial Surg..

[152] Meyer D.R. (1995). Alloplastic materials for orbital surgery. Curr. Opin. Ophthalmol..

[153] Ozturk S., Sengezer M., Isik S., Turegun M., Deveci M., Cil Y. (2005). Long-term outcomes of ultra-thin porous polyethylene implants used for reconstruction of orbital floor defects. J. Craniofac. Surg..

[154] Tercan M. (1995). Thin Silastic sheet for orbital floor repair. Plast. Reconstr. Surg..

[155] Xu J., Teng L., Jin X., Ji Y., Lu J., Zhang B. (2009). Porous polyethylene implants in orbital blow-out fractures and enophthalmos reconstruction. J. Craniofac. Surg..

[156] Lemke B.N., Kikkawa D.O. (1999). Repair of orbital floor fractures with hydroxyapatite block scaffolding. Ophthal. Plast. Reconstr. Surg..

[157] Teo L., Teoh S.H., Liu Y., Lim L., Tan B., Schantz J.-T., Seah L.L. (2015). A Novel Bioresorbable Implant for Repair of Orbital Floor Fractures. Orbit Amst. Neth..

[158] Gierloff M., Seeck N.G.K., Springer I., Becker S., Kandzia C., Wiltfang J. (2012). Orbital floor reconstruction with resorbable polydioxanone implants. J. Craniofac. Surg..

[159] Burres S.A., Cohn A.M., Mathog R.H. (1981). Repair of orbital blowout fractures with marlex mesh and gelfilm. Laryngoscope.

[160] Young S.M., Sundar G., Lim T.-C., Lang S.S., Thomas G., Amrith S. (2017). Use of bioresorbable implants for orbital fracture reconstruction. Br. J. Ophthalmol..

[161] Tsumiyama S., Umeda G., Ninomiya K., Miyawaki T. (2019). Use of Unsintered Hydroxyapatite and Poly-L-lactic Acid Composite Sheets for Management of Orbital Wall Fracture. J. Craniofac. Surg..

[162] Kohyama K., Morishima Y., Arisawa K., Arisawa Y., Kato H. (2018). Immediate and long-term results of unsintered hydroxyapatite and poly L-lactide composite sheets for orbital wall fracture reconstruction. J. Plast. Reconstr. Aesthet. Surg..

[163] Watanabe A., Yamanaka Y., Rajak S.N., Nakayama T., Ueda K., Sotozono C. (2021). Assessment of a Consecutive Series of Orbital Floor Fracture Repairs With the Hess Area Ratio and the Use of Unsintered Hydroxyapatite Particles/Poly l-Lactide Composite Sheets for Orbital Fracture Reconstruction. J. Oral Maxillofac. Surg..

[164] Jang H.U., Kim S.Y. (2020). Biodegradable implants for orbital wall fracture reconstruction. Arch. Craniofacial Surg..

[165] Kanno T., Karino M., Yoshino A., Koike T., Ide T., Tatsumi H., Tsunematsu K., Yoshimatsu H., Sekine J. (2017). Feasibility of Single Folded Unsintered Hydroxyapatite Particles/Poly-L-Lactide Composite Sheet in Combined Orbital Floor and Medial Wall Fracture Reconstruction. J. Hard Tissue Biol..

[166] Kanno T., Tatsumi H., Karino M., Yoshino A., Koike T., Ide T., Sekine J. (2016). Clinical Report: Applicability of an Unsintered Hydroxyapatite Particles/Poly-L-Lactide Composite Sheet with Tack Fixation for Orbital Fracture Reconstruction. J. Hard Tissue Biol..

[167] Sukegawa S., Kanno T., Koyama Y., Matsumoto K., Sukegawa-Takahashi Y., Masui M., Tanaka S., Furuki Y. (2017). Precision of Post-Traumatic Orbital Reconstruction Using Unsintered Hydroxyapatite Particles/Poly-L-Lactide Composite Bioactive/Resorbable Mesh Plate with and without Navigation: A Retrospective Study. J. Hard Tissue Biol..

[168] Shintaro S., Takahiro K., Yuta K., Akane S., Ken-ichi M., Yuka S., Kyosuke S., Shigeto T., Yoshihiko F. (2017). Intraoperative Navigation-assisted Surgical Orbital Floor Reconstruction in Orbital Fracture Treatment: A Case Report. Shimane J. Med. Sci..

[169] Dong Q.N., Karino M., Koike T., Ide T., Okuma S., Kaneko I., Osako R., Kanno T. (2020). Navigation-Assisted Isolated Medial Orbital Wall Fracture Reconstruction Using an U-HA/PLLA Sheet via a Transcaruncular Approach. J. Investig. Surg..

[170] Dong Q.N., Ide T., Karino M., Okuma S., Koike T., Kanno T. (2019). Retrobulbar Orbital Emphysema Associated with Medial Orbital Wall Fracture. J. Craniofac. Surg..

[171] Hwang K. (2019). In Vivo Degradation of Forged-Unsintered Hydroxyapatite and Poly-L-lactide Mesh Used for Orbital Reconstruction. J. Craniofac. Surg..

[172] Hayashi M., Muramatsu H., Sato M., Tomizuka Y., Inoue M., Yoshimoto S. (2013). Surgical treatment of facial fracture by using unsintered hydroxyapatite particles/poly l-lactide composite device (OSTEOTRANS MX(^®^)): A clinical study on 17 cases. J. Cranio-Maxillofacial Surg..

[173] Eppley B.L., Sadove A.M. (1994). Effects of resorbable fixation on craniofacial skeletal growth: Modifications in plate size. J. Craniofac. Surg..

[174] Eppley B.L., Sparks C., Herman E., Edwards M., McCarty M., Sadove A.M. (1993). Effects of skeletal fixation on craniofacial imaging. J. Craniofac. Surg..

[175] Yu J.C., Bartlett S.P., Goldberg D.S., Gannon F., Hunter J., Habecker P., Whitaker L.A. (1996). An experimental study of the effects of craniofacial growth on the long-term positional stability of microfixation. J. Craniofac. Surg..

[176] Alpert B., Seligson D. (1996). Removal of asymptomatic bone plates used for orthognathic surgery and facial fractures. J. Oral Maxillofac. Surg..

[177] Kim Y.M., Lee J.H. (2019). Clinical courses and degradation patterns of absorbable plates in facial bone fracture patients. Arch. Craniofacial Surg..

[178] Lee S.J., Park E.S., Nam S.M., Choi C.Y., Shin H.S., Kim Y.B. (2019). Surgical Treatment of Mandible Fracture Using Unsintered Hydroxyapatite/Poly L-Lactide Composite Fixation System. J. Craniofac. Surg..

[179] Song I.-S., Choi J., Kim S.R., Lee J.-H. (2019). Stability of bioresorbable plates following reduction of mandibular body fracture: Three-dimensional analysis. J. Cranio-Maxillofacial Surg..

[180] Kellman R.M., Cienfuegos R. (2009). Endoscopic approaches to subcondylar fractures of the mandible. Facial Plast. Surg. FPS.

[181] Son J.-H., Ha J., Cho Y.-C., Sung I.-Y. (2017). Are Biodegradable Plates Applicable in Endoscope-Assisted Open Reduction and Internal Fixation of Mandibular Subcondyle Fractures?. J. Oral Maxillofac. Surg..

[182] Kim D.-Y., Sung I.-Y., Cho Y.-C., Park E.-J., Son J.-H. (2018). Bioabsorbable plates versus metal miniplate systems for use in endoscope-assisted open reduction and internal fixation of mandibular subcondylar fractures. J. Cranio-Maxillofacial Surg..

[183] Sukegawa S., Kanno T., Yamamoto N., Nakano K., Takabatake K., Kawai H., Nagatsuka H., Furuki Y. (2019). Biomechanical Loading Comparison between Titanium and Unsintered Hydroxyapatite/Poly-L-Lactide Plate System for Fixation of Mandibular Subcondylar Fractures. Materials.

[184] Sukegawa S., Yamamoto N., Nakano K., Takabatake K., Kawai H., Kanno T., Nagatsuka H., Furuki Y. (2020). Biomechanical Loading Comparison between Titanium and Bioactive Resorbable Screw Systems for Fixation of Intracapsular Condylar Head Fractures. Materials.

[185] Jorgenson D.S., Mayer M.H., Ellenbogen R.G., Centeno J.A., Johnson F.B., Mullick F.G., Manson P.N. (1997). Detection of titanium in human tissues after craniofacial surgery. Plast. Reconstr. Surg..

[186] Haers P.E., Sailer H.F. (1998). Biodegradable self-reinforced poly-L/DL-lactide plates and screws in bimaxillary orthognathic surgery: Short term skeletal stability and material related failures. J. Cranio-Maxillofacial Surg..

[187] Cheung L.K., Chow L.K., Chiu W.K. (2004). A randomized controlled trial of resorbable versus titanium fixation for orthognathic surgery. Oral Surg. Oral Med. Oral Pathol. Oral Radiol. Endod..

[188] Landes C.A., Ballon A. (2006). Skeletal stability in bimaxillary orthognathic surgery: P(L/DL)LA-resorbable versus titanium osteofixation. Plast. Reconstr. Surg..

[189] Landes C.A., Ballon A., Sader R. (2007). Segment stability in bimaxillary orthognathic surgery after resorbable Poly(L-lactide-co-glycolide) versus titanium osteosyntheses. J. Craniofac. Surg..

[190] Stockmann P., Böhm H., Driemel O., Mühling J., Pistner H. (2010). Resorbable versus titanium osteosynthesis devices in bilateral sagittal split ramus osteotomy of the mandible—The results of a two centre randomised clinical study with an eight-year follow-up. J. Cranio-Maxillofacial Surg..

[191] Matthews N.S., Khambay B.S., Ayoub A.F., Koppel D., Wood G. (2003). Preliminary assessment of skeletal stability after sagittal split mandibular advancement using a bioresorbable fixation system. Br. J. Oral Maxillofac. Surg..

[192] Ueki K., Okabe K., Moroi A., Marukawa K., Sotobori M., Ishihara Y., Nakagawa K. (2012). Maxillary stability after Le Fort I osteotomy using three different plate systems. Int. J. Oral Maxillofac. Surg..

[193] Okabe K., Ueki K., Marukawa K., Mukozawa A., Miyazaki M., Nakagawa K. (2010). An experimental study of use of absorbable plate in combination with self-setting α-tricalcium phosphate for orthognathic surgery. Oral Surg. Oral Med. Oral Pathol. Oral Radiol. Endod..

[194] Ueki K., Okabe K., Marukawa K., Mukozawa A., Moroi A., Miyazaki M., Sotobori M., Ishihara Y., Yoshizawa K., Ooi K. (2013). Maxillary stability after Le Fort I osteotomy with self-setting α-tricalcium phosphate and an absorbable plate. Int. J. Oral Maxillofac. Surg..

[195] Ueki K., Yoshizawa K., Moroi A., Hotta A., Tsutsui T., Fukaya K., Hiraide R., Takayama A., Tsunoda T., Saito Y. (2017). Evaluation of maxillary sinus after Le Fort I osteotomy using various fixation materials. J. Cranio-Maxillofacial Surg..

[196] Meningaud J.-P., Poupon J., Bertrand J.-C., Chenevier C., Galliot-Guilley M., Guilbert F. (2001). Dynamic study about metal release from titanium miniplates in maxillofacial surgery. Int. J. Oral Maxillofac. Surg..

[197] Siniscalchi E.N., Catalfamo L., Allegra A., Musolino C., De Ponte F.S. (2013). Titanium miniplates: A new risk factor for the development of the bisphosphonate-related osteonecrosis of the jaw. J. Craniofac. Surg..

[198] Ueki K., Okabe K., Miyazaki M., Mukozawa A., Moroi A., Marukawa K., Nakagawa K., Yamamoto E. (2011). Skeletal stability after mandibular setback surgery: Comparisons among unsintered hydroxyapatite/poly-L-lactic acid plate, poly-L-lactic acid plate, and titanium plate. J. Oral Maxillofac. Surg..

[199] Chung I.-H., Yoo C.-K., Lee E.-K., Ihm J.-A., Park C.-J., Lim J.-S., Hwang K.-G. (2008). Postoperative Stability After Sagittal Split Ramus Osteotomies for a Mandibular Setback With Monocortical Plate Fixation or Bicortical Screw Fixation. J. Oral Maxillofac. Surg..

[200] Van Sickels J.E. (1991). A comparative study of bicortical screws and suspension wires versus bicortical screws in large mandibular advancements. J. Oral Maxillofac. Surg..

[201] Brasileiro B.F., Grotta-Grempel R., Ambrosano G.M.B., Passeri L.A. (2012). An in vitro evaluation of rigid internal fixation techniques for sagittal split ramus osteotomies: Setback surgery. J. Oral Maxillofac. Surg..

[202] Schwartz H.C., Relle R.J. (1996). Bicortical-monocortical fixation of the sagittal mandibular osteotomy. J. Oral Maxillofac. Surg..

[203] Ueki K., Okabe K., Marukawa K., Mukozawa A., Moroi A., Miyazaki M., Sotobori M., Ishihara Y., Yoshizawa K., Ooi K. (2014). Skeletal stability after mandibular setback surgery: Comparison between the hybrid technique for fixation and the conventional plate fixation using an absorbable plate and screws. J. Cranio-Maxillofacial Surg..

[204] Ueki K., Moroi A., Yoshizawa K., Hotta A., Tsutsui T., Fukaya K., Hiraide R., Takayama A., Tsunoda T., Saito Y. (2017). Comparison of skeletal stability after sagittal split ramus osteotomy among mono-cortical plate fixation, bi-cortical plate fixation, and hybrid fixation using absorbable plates and screws. J. Cranio-Maxillofacial Surg..

[205] Ueki K., Moroi A., Ishihara Y., Sotobori M., Iguchi R., Kosaka A., Ikawa H., Yoshizawa K., Marukawa K. (2014). Comparison of lower lip hypoesthesia between hybrid fixation and conventional fixation following sagittal split ramus osteotomy. J. Cranio-Maxillofacial Surg..

[206] Ueki K., Moroi A., Iguchi R., Kosaka A., Ikawa H., Yoshizawa K. (2015). Changes in the computed tomography (pixel) value of mandibular ramus bone and fixation screws after sagittal split ramus osteotomy. Int. J. Oral Maxillofac. Surg..

[207] Yamashita Y., Mizuashi K., Shigematsu M., Goto M. (2007). Masticatory function and neurosensory disturbance after mandibular correction by bilateral sagittal split ramus osteotomy: A comparison between miniplate and bicortical screw rigid internal fixation. Int. J. Oral Maxillofac. Surg..

[208] Sukegawa S., Kanno T., Manabe Y., Matsumoto K., Sukegawa-Takahashi Y., Masui M., Furuki Y. (2017). Biomechanical Loading Evaluation of Unsintered Hydroxyapatite/poly-l-lactide Plate System in Bilateral Sagittal Split Ramus Osteotomy. Materials.

[209] Park Y.-W., Kang H.-S., Lee J.-H. (2019). Comparative study on long-term stability in mandibular sagittal split ramus osteotomy: Hydroxyapatite/poly-l-lactide mesh versus titanium miniplate. Maxillofac. Plast. Reconstr. Surg..

[210] Takayama A., Moroi A., Saito Y., Yoshizawa K., Nishida T., Ueki K. (2019). Evaluation of Space-Maintaining Sinus Membrane Using the Absorbable Screws in Sinus Lifting Bone Augmentation. Implant Dent..

[211] Tatsuta S., Hayashi M., Tokunaka R., Muramatsu H., Kadomatsu K. (2018). Analysis of the Postoperative Absorption Process of Unsintered Hydroxyapatite Particles/Poly L-Lactide Composite Device (OSTEOTRANS MX^®^) for Facial Bone Fractures in 13 Cases. Clin. Surg..

